# Microbial composition differs between production systems and is associated with growth performance and carcass quality in pigs

**DOI:** 10.1186/s42523-021-00118-z

**Published:** 2021-08-28

**Authors:** Christian Maltecca, Rob Dunn, Yuqing He, Nathan P. McNulty, Constantino Schillebeeckx, Clint Schwab, Caleb Shull, Justin Fix, Francesco Tiezzi

**Affiliations:** 1 Department of Animal Science, North Carolina State University, 120 W Broughton Dr, Raleigh, NC 27607 USA; 2 Department of Applied Ecology, North Carolina State University, 100 Brooks Ave, Raleigh, NC 27607 USA; 3 Matatu Inc., 4340 Duncan Ave Suite 211, St. Louis, MO 63110 USA; 4 Acuity Ag Solutions, 7475 State Route 127, Carlyle, IL 62231 USA; 5 The Maschhoffs LLC, 7475 IL-127, Carlyle, IL 62231 USA

**Keywords:** Microbiome, Microbial diversity, Growth, Host genetics, Production system, Carcass quality, Swine

## Abstract

**Background:**

The role of the microbiome in livestock production has been highlighted in recent research. Currently, little is known about the microbiome's impact across different systems of production in swine, particularly between selection nucleus and commercial populations. In this paper, we investigated fecal microbial composition in nucleus versus commercial systems at different time points.

**Results:**

We identified microbial OTUs associated with growth and carcass composition in each of the two populations, as well as the subset common to both. The two systems were represented by individuals with sizeable microbial diversity at weaning. At later times microbial composition varied between commercial and nucleus, with species of the genus *Lactobacillus* more prominent in the nucleus population. In the commercial populations, OTUs of the genera *Lactobacillus* and *Peptococcus* were associated with an increase in both growth rate and fatness. In the nucleus population, members of the genus *Succinivibrio* were negatively correlated with all growth and carcass traits, while OTUs of the genus *Roseburia* had a positive association with growth parameters. *Lactobacillus* and *Peptococcus* OTUs showed consistent effects for fat deposition and daily gain in both nucleus and commercial populations. Similarly, OTUs of the *Blautia* genus were positively associated with daily gain and fat deposition. In contrast, an increase in the abundance of the *Bacteroides* genus was negatively associated with growth performance parameters.

**Conclusions:**

The current study provides a first characterization of microbial communities' value throughout the pork production systems. It also provides information for incorporating microbial composition into the selection process in the quest for affordable and sustainable protein production in swine.

**Supplementary Information:**

The online version contains supplementary material available at 10.1186/s42523-021-00118-z.

## Background

The microbiota, the community of bacteria, viruses, and microbial eukaryotes that live on and in other organisms, is increasingly recognized for the role they play in altering host phenotype [1]. The microbiota mediates an organism’s relationship to its environment through their dual effects on host phenotype: the genomic repertoire of microbes serves as an extension to that of the host and signaling from the microbiota can alter host functioning [2]. Host traits relevant to areas as diverse as metabolism, immunity, physiology, and behavior have all been linked to the gut microbiota [3–5]. Today, microbiota-based interventions are being developed to improve host wellbeing in aspects as diverse as infectious disease [6], productivity [7], and conservation [8]. In agriculture, research has chiefly focused on the nutritional effects of the microbiota and probiotic or prebiotic interventions to improve animal condition [9, 10]. Previous research [11–13] has highlighted how animals with disrupted microbiota or reduced microbial diversity have an increased risk of obesity as well as several other diseases.

This symbiotic relationship between host and gut microbes is also relevant in pigs [14]. The gut microbiome unlocks energy from undigested feed components through fermentation. Simultaneously, it provides a barrier that restricts pathogen invasions and complements the protective function of the host immune system [15]. Along with metabolizing various foods, the microbiota provides vitamins B and K and indole derivatives [16, 17]. These components help in the intestine’s growth and development and improve the absorption of nutrients [18].

Despite increased interest in the gut microbiota and its potential agricultural applications, much remains unknown about host-microbe interactions and their impact on host productivity in pigs. Recent studies have documented associations between the microbiota and various environmental and management parameters [19, 20]. In previous research from our group, we have shown how fecal microbiota diversity can be used as an indicator trait to improve efficiency traits that are expensive to measure [21]. We have further demonstrated how microbiome composition can effectively be used as a predictor of growth and carcass composition traits [22] as well as how differences in gut microbial composition throughout the growth period of different breeds of pigs shape feed efficiency within and across breed [23]. Most recently, we identified heritable pig gut microbiome OTUs associated with growth and fatness and putative host genetic markers associated with significant differences in the abundance of several prevalent microbiome features [24]. Despite these efforts, several limitations plague current research efforts in understanding the interconnections between the host and its microbiome. Most notably, the inability to transfer results from different populations and conditions is due to the use of small and relatively disconnected experiments [25].

Nucleus and commercial systems represent different environments within the pork industry. The industry makes extensive use of crossbreeding to leverage genetic complementarity among breeds and hybrid vigor. Typically in an integrated swine system, two purebred genetic lines are crossed to obtain F1 individuals. Females of these crosses are then mated to a third breed to generate three-way crossbred pigs. All crossbred pigs are sent to market, while the originating elite purebred individuals are used as breeders of subsequent generations. Thanks to this system, the high prolificacy, and the species’ short generation interval, a few thousand purebred individuals can generate millions of crossbred individuals destined for the market. Additionally, as a result of this structure, purebred and crossbred individuals are kept at different farms throughout their life. This is because purebred individuals carry a higher economic value, and thus stricter biosecurity protocols are employed at purebred nucleus facilities. This leads to a different microbial composition of the nucleus vs. commercial environments, potentially reflecting on gut microbial composition differences. To date, little is known about the microbiome’s impact across these different systems of pork production.

In this paper, we compare gut microbial composition over time in nucleus versus commercial systems to understand the gut microbiota and its contribution to swine production. Specifically, we compare the overall ecology of the two setups by identifying taxa differentially represented across time points and systems. We further investigated the existence of cluster of individuals based on their taxonomical abundance among the two systems. Finally, we identify microbial OTUs related to growth and carcass composition characteristics of each of the systems and in common among the two.

## Methods

### Experimental design and data collection

Phenotypic records presented in this study came from a commercial and a nucleus farm operated by The Maschhoffs LLC (Carlyle, IL, USA). All methods and procedures followed the Animal Care and Use policies of North Carolina State University and the National Pork Board. The experimental protocol for fecal sample collection received approval number 15027 from the Institutional Animal Care and Use Committee. All pigs were harvested in commercial facilities under the supervision of the USDA Food Safety and Inspection Service.

The data spanned two connected populations/trials: a Duroc nucleus purebred population **(NU)** and a terminal commercial crossbred population **(TE)**, both sired by 28 Duroc founding boars. Identification, sex, cross-fostering status, litter, and sow identification and parity were collected for all individuals in the experiment.

The NU population consisted of 819 Duroc individuals (males and females). Individuals were raised under controlled conditions typical of nucleus farms in a *fixed-time* system. Individuals were put on test at 88.5 ± 9.92 days of age and taken off-test at 178.4 ± 7.96 days of age (average 129.49 ± 17.72 kg of weight). The TE population consisted of 1 257 individuals (females and castrated males) generated by crossing the Duroc sires with two commercial sow lines (Yorkshire x Landrace and Landrace x Yorkshire) lines. Crossbred commercial individuals were raised in a *fixed-weight* testing system (similar to most commercial operations) and harvested at an average weight of 98.8 ± 10.19 kg and 97.9 ± 7.63 kg for the males and females, respectively. Throughout the experiment, and in both systems, a contemporary group was defined as the group of animals that entered a given facility at the same time. For both systems, individuals were allocated in single-sire, single-sex groups of twenty heads and housed in the same pen. Feed and water were provided ad libitum to pigs. Details of diets and their nutritional values are provided in Additional file 1. The pigs received a standard vaccination and medication routine (Additional file 2). Rectal swabs were collected from all pigs at three time points: weaning (**TP1**; as described above for NU, average 90.63 ± 1.57 days for TE), mid-test (**TP2**; average 118.2 ± 1.18 days for TE and 116.3 ± 2.3 for NU), and off-test (**TP3**; as described above for NU and 176.45 ± 1.82 days for TE). In both systems, four to five pigs from each pen were selected as detailed by (Wilson et al., 2016). The pigs selected for each pen represented an average pig for body weight, along with pigs approximately 1 and 2 SD above and below the pen average. Their rectal swabs were used for subsequent microbial sequencing.

There were a total of 1 205 and 803, 1 295 and 811, 1 282 and 824 samples, collected at TP1, TP2, and TP3, in TE and NU, respectively.

### Microbial sequences bioinformatics and processing

#### 16S rRNA gene sequencing

DNA extraction, purification, Illumina library preparation, and sequencing were done as described by Lu and colleagues [21]. Briefly, total DNA (gDNA) was extracted from each rectal swab by mechanical disruption in phenol:chloroform:isoamyl alcohol solution. Bead-beating was performed on the Mini-BeadBeater-96 (MBB-96; BioSpec, OK, USA) for 4 min at room temperature, and samples were centrifuged at 3 220 × *g*. The DNA was then purified using a QIAquick 96 PCR purification kit (Qiagen, MD, USA), with minor modifications to the manufacturer’s protocol. Modifications included the addition of sodium acetate (3 M, pH 5.5) to Buffer PM to a final concentration of 185 mM, combining crude DNA with four volumes of Buffer PM, and elution of DNA in 100 µL of Buffer EB. All sequencing was performed at the DNA Sequencing Innovation Laboratory at the Center for Genome Sciences & Systems Biology at Washington University in St. Louis. Phased, bi-directional amplification of the V4 region (bases 515–806) of the 16S rRNA gene was employed to generate indexed libraries for Illumina sequencing as described in Faith et al. [26]. Sequencing was performed on an Illumina MiSeq instrument (Illumina, Inc. San Diego, USA), generating 250 bp paired-end reads.

#### Taxonomic classification

16S rRNA gene sequencing and quality control of the data were conducted as described by Lu and colleagues [21]. Briefly, the pairs of 16S rRNA gene sequences obtained from Illumina sequencing were combined into single sequences using FLASH v1.2.11 [27]. The sequences with a mean quality score below Q35 were filtered out using PRINSEQ v0.20.4 [28]. Forward-oriented sequences were searched for primer sequences, allowing up to 1 bp of mismatch, and primer sequences were trimmed. Sequences were subsequently demultiplexed using QIIME v1.9 [29].

QIIME was used to cluster the nucleotide sequences into operational taxonomic units (OTUs) using open-reference OTU picking as described by Lu et al. [21]. A modified version of GreenGenes [30, 31] was used as the reference database. Then, the 90% of reads matched with the reference database were assigned to the new reference OTU derived from the de novo cluster. Sparse OTUs with fewer than 1 200 total observed counts were subsequently removed. Finally, the Ribosomal Database Project (RDP) classifier (v2.4) was retrained in the manner described in [32], and a bootstrap cutoff value of 0.8 was used to assign taxonomy to the representative sequences. The resulting OTU table was rarefied to 10 000 counts per sample, and 3 001 OTUs were retained for further analyses.

Metagenomic predictions were obtained using PICRUSt [33]. Second-level and third-level ontology pathways of the Kyoto Encyclopedia of Genes and Genomes [34] were obtained using the *categorize_by_function* and the *metagenome_contribution* functions.

The table of individual OTU counts, along with their metadata and taxonomic classifications, was merged into a single object of class *phyloseq* in R [35]. The same package was used for several of the subsequent analyses.

#### Diversity analyses

Alpha diversity analyses were conducted with univariate linear regression models. Diversity metrics were obtained via the *phyloseq* package using the *estimate_richness* function and included: observed richness, Inverse Simpson, Shannon index, and Chao1 index. To test the significance of experimental features, the *lm* package in R [36] was used. Least-squares-means were obtained using the *pairwise* option with p-value adjustment of Tukey in the *lsmeans* function of the *emmeans* package [37]. Factors included in the analysis were: sire, contemporary group (within system), sex (within system), age at sampling (TP1, TP2, TP3), system (NU or TE), plus the interaction between system and age at sampling, sire and age at sampling, and system and sire.

Cluster analysis was performed as described by Arumugam et al. [38]. Samples were clustered using the Jensen-Shannon divergence (JSD) distance and Partitioning Around Medoids (PAM) clustering using the function *pam* of the package *cluster* in R [39]. To determine the optimal number of clusters, the gap statistic [40] was evaluated from 2 to 8 clusters using the function *clusGap* of the package *cluster* in R. The gap statistic compares the total intra-cluster variation for different number of clusters with their expected values under uniform distribution of the data. The optimal cluster number is the value that maximizes the gap statistic. In the analysis the final number of clusters was determined by visual inspection of the increase in the gap statistics. The number of clusters as the smallest value of *k* (the cluster number) such that the gap statistic was within one standard deviation of the gap at *k* + 1.

Feature importance (the ability of a feature to discriminate a cluster) at each time was evaluated using the mean decrease in Gini index after applying a Random Forest algorithm as implemented in the package *caret* in R [41].

Between-sample (beta) diversity was assessed using the Bray–Curtis distance dissimilarity metric [42]. Permutational multivariate analysis of variance (PERMANOVA) using the *adonis* function of the R package *vegan* [43] with 5 000 permutations was performed to analyze the distances dissimilarities for the system, sire, and sex factors for each of the three ages considered.

Differential abundance of OTUs at different time points among systems was obtained through a negative binomial model implemented through the package *DESeq2* [44] in R. The model included the effect of sire, system, age, sex, and contemporary group. Contrasts were obtained for the system effect (NU vs. TE) for each of the three sampling times. The significance of each contrast was assessed using the Wald Chi-Squared Test.

#### Association of microbial OTUs with growth and carcass composition in nucleus and commercial systems

The association between microbial OTUs and the traits of interest was performed independently for the two systems. This was dictated assuming that both the genotype and the environment would affect the gut microbiota [24]. The microbial covariates included OTU relative abundance and second-level ontology pathways of the Kyoto Encyclopedia of Genes and Genomes [34]. Before the association analysis, the microbial covariates were treated using Bayesian-Multiplicative replacement of zero counts using the *cmultRepl* function from the R package *zCompositions* [45] and centered log-ratio transformation using the function *clr* from the R package *compositions* [46]. The OTUs relative abundance and KEGG pathways representation were considered as different variables according to the sampling stage. The 3 001 OTUs therefore became 9 003 independent covariates, and the 39 identified pathways became 117 independent covariates.

#### Terminal commercial system

The phenotypes used in the association analysis for TE were the same of Khanal et al. [47, 48]. Briefly, carcass quality traits included measures of body growth and tissue deposition taken at harvest (TP3), such as carcass average daily gain (**cADG**) as the eviscerated body weight accumulated from birth to harvest; loin depth (**cLD**) as the depth of the loin muscle; back-fat depth (**cBF**) as the depth of the fat layer in correspondence of the 10^th^ thoracic vertebra; ham yield (**cHAM**), loin yield (**cLOI**), belly yield (**cYEL**) as the proportion of the ham, loin and belly cuts on carcass weight, respectively. Meat quality traits included subjective (sensory panel assessed) measures of color (**cSCOL**), firmness (**cSFIR**) and marbling (**cSMAR**) as well as objective measures of color (**cMinL**, **cMinA** and **cMinB**), intra-muscular fat deposition (**cIMF**) and firmness (**cSSF**). Meat quality traits also included muscle pH recorded after *rigor mortis* (**cPH**).

The association was conducted fitting a series of linear mixed models that sequentially included the linear effect of the microbial covariate. In addition, other effects were fit as dictated by the experimental design. The linear mixed model formula was:

$$y_{ijklmn} = \mu + Micro_{i} + Sire_{j} + CG_{k} + DL_{l} + pen_{m} + e_{ijklmn}$$

where $y_{ijklm}$ is a vector of phenotypic values; $Micro_{i}$ is the linear effect of one of the microbial covariates (an OTUs or pathways representation), $Sire_{j}$ is the effect of the j-th sire (28 levels), $CG_{k}$ is the effect of the k-th contemporary group (12 levels), $DL_{l}$ is the fixed effect of the maternal genetic line (2 levels), $pen_{m}$ is the random effect of the physical group of same-sex paternal-half-sibs individuals and $e_{ijklmn}$ is the residual error. The model was fitted using the function $lmer$ of the R package $lme4$ [49]. Significance of the microbial effect was assessed calculating one-tailed p-value using the estimate and the standard error of the regression coefficient, false discovery rate adjustment (function p.adjust in R) was performed and only effects with an adjusted p-value smaller than 0.05 were considered significant. The proportion of phenotypic variance absorbed was calculated as the ratio between the variance absorbed and the total phenotypic variance of the traits. The variance absorbed was calculated as the variance of the vector obtained multiplying the regression coefficient by the microbial covariate vector.

#### Nucleus system

The phenotypes used in this analysis were recorded at the end of the performance test. Traits included: body weight (**pBW**), loin muscle depth (**pLD**) and area (**pLA**); back-fat depth (**pBF**) and loin intra-muscular fat concentration (**pIMF**). In addition, average body weight daily gain from birth to the end of test (**pADG**) was calculated as the difference between NUW and birth weight and divided by the age of the individual at the end of the performance test. Traits pLD, pLA, pBF and pIMF were obtained using an ultrasound probe as in Bergamaschi et al. [50].

As for TE, the association in the NU population was conducted by fitting a series of linear mixed models that sequentially included the linear effect of the microbial covariate in addition to the other effects as dictated by the experimental design. The linear mixed model formula was:

$$y_{ijklmn} = \mu + Micro_{i} + Sire_{j} + CG_{k} + Sex_{l} + Litter_{m} + e_{ijklmn}$$

where $y_{ijklmn}$ was a vector of phenotypic values; $Micro_{i}$ is the linear effect of one of the microbial covariates (an OTU or pathways representation), $Sire_{j}$ is the effect of the j-th sire (28 levels), $CG_{k}$ is the effect of the k-th contemporary group (66 levels), $Sex_{l}$, is the effect of sex (2 levels), $Litter_{m}$ is the random effect of the biological litter where the individual was born and $e_{ijklmn}$ is the residual error. Model fitting as well as significance and proportion of variance explained by the microbial effect were obtained as for the TE population.

## Results

### Taxonomic abundance

We obtained 6 223 fecal samples (2 442 NU; 3 781 TE) from a total of 2 076 individual pigs (1 257 TE; 819 NU). Of 2 076 pigs, 1 846 had complete observations for all three sampling points (1 039 TE; 807 NU).

**Fig. 1:**
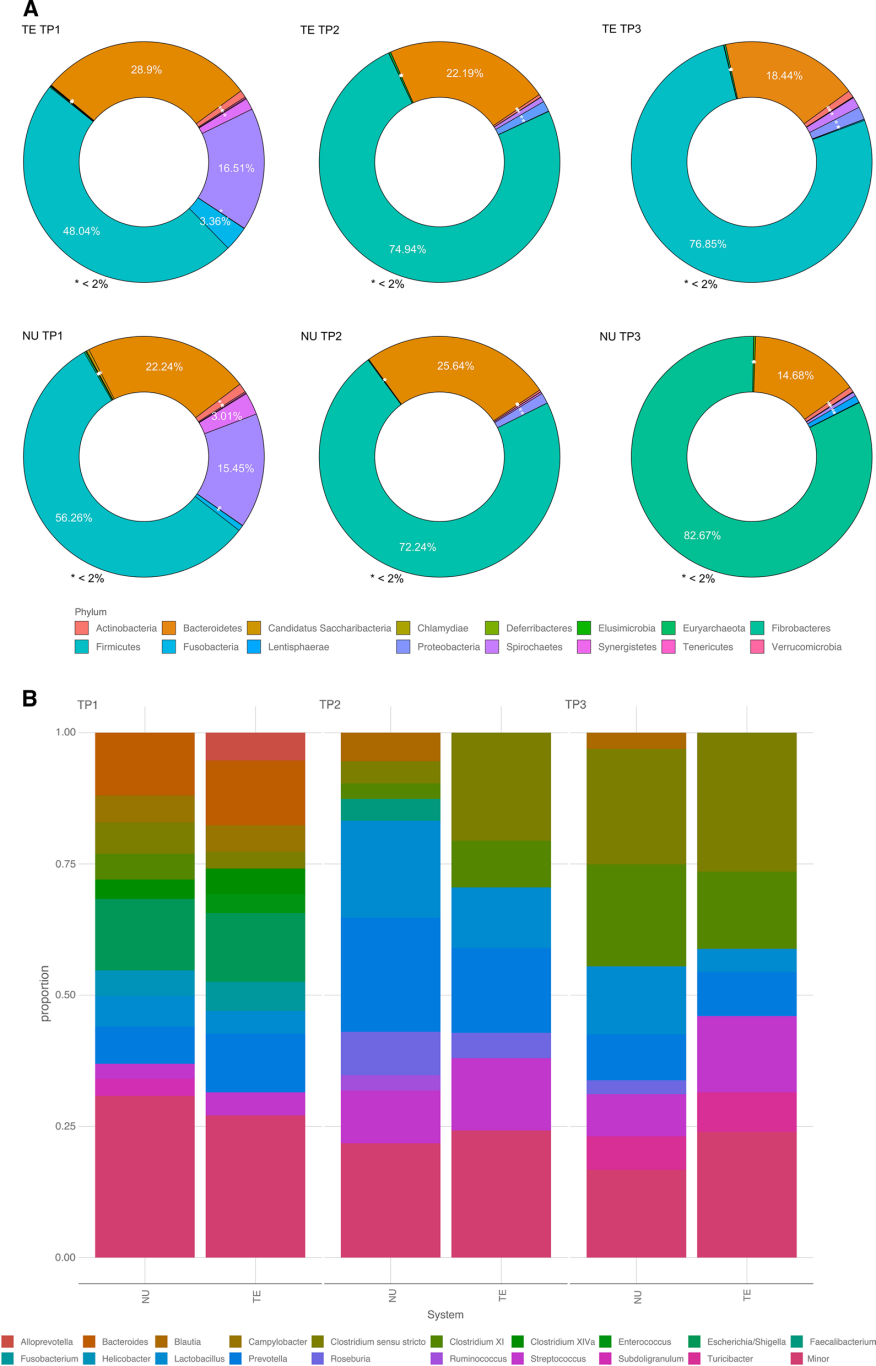
Relative abundance of microbiome taxa for two systems at three time points. Relative abundance of microbiome taxa at Phylum (**a**) and Genus level (**b**) of Purebred (NU) and Crossbred (TE) at three time points: weaning (TP1), mid test (TP2), and off test (TP3) of the feeding trial

Across both sampled systems, 75.6% and 41.55% of the total sequences were assigned to 16 phyla and 129 genera, respectively. Firmicutes and Bacteroidetes constituted the two predominant phyla in the fecal microbiota of pigs (contributing 68.4 and 22.2% of the total classified sequences, respectively) across systems and time. These were followed by Proteobacteria (6.2%) and Spirochaetes (1.2%). When data were stratified by timepoint, the diversity of bacterial phyla decreased through time. At TP1, Firmicutes were relative abundant, comprising 51.3% of sequences (48.04% TE; 56.6% NU; Fig. 1a). Bacteroidetes represented 26.2% of sequences (28.9% TE; 22.24% NU; Fig. 1a). The third most frequent phylum was Proteobacteria representing 16.5% of TE and 15.4% of NU sequences. With regard to the number of sequences, Fusobacteria was the fourth most abundant phylum in TE 3.36%, while Spirochaetes were the fourth most abundant in NU with 3.0% of the represented sequences. At time points two and three, the proportion of sequences from Firmicutes increased both in TE (74.9%/76.8%) and NU (72.2%/82.2%). Conversely, the Bacteroidetes representation decreased in TE (22.2%/18.4%) and NU (25.6%/14.9%) at time points two and three. At TP3, nearly all reads were from either Firmicutes or Bacteroidetes (95.3% in TE; 97.3% in NU).

At the genus level, 27 taxa accounted for ~ 90% of the total assigned sequences across systems and time (Fig. 1b). *Clostridium *sensu stricto (14.9%), *Prevotella* (12.4%), *Streptococcus* (9.6%), *Lactobacillus* (9.3%), and *Clostridium* XI (8.8%) were the 5 most abundant genera. When parceling the results by time, at TP1, *Escherichia/Shigella* was the most abundant genus (13.1% TE; 13.6% NU), followed by *Bacteroides* and *Prevotella* (12.4%, 11.2% and 11.9%, 7.1%, for TE and NU). The fourth most abundant genus in TE was *Fusobacterium* (5.5%) while it was *Clostridium *sensu stricto (5.93%) in NU; these were followed by *Alloprevotella* (5.3%) and *Lactobacillus* (5.9%) for TE and NU, respectively. At TP2, the five most abundant genera in TE were *Clostridium *sensu stricto (20.6%), *Prevotella* (16.2%), *Streptococcus* (13.8%), *Lactobacillus* (11.5%), and *Clostridium XI* (8.9%). In contrast, for NU the most abundant were *Prevotella* (21.7%), *Lactobacillus* (18.5%), *Streptococcus* (10.0%), *Roseburia* (9.2%) and *Blautia* (5.4%). At TP3, nine of the most represented 10 genera were in common amongst TE and NU. The top five were: *Costridium *sensu* strictu* (26.5% TE; 22.0% NU); *Clostridium XI* (14.7% TE; 19.4% NU); *Streptococcus* (14.5% TE; 8.1% NU); *Prevotella* (8.4% TE; 8.7% NU), and *Turicibacter* (7.5% TE; 6.4% NU). Interestingly, the genus *Lactobacillus* was notably more present in NU (13.0%), than in TE (4.4%). In general, both at the begining and the end of the trial, TE and NU had a similar microbial composition regarding genera, while they were more discrepant at TP2 (Fig. 1b). The key differences at TP2 were *Turicibacter*, *Clostridium* XI, and *Faecalibacterium* between the two systems.

### Pathways abundance

The relative abundance of different metabolic pathways for the two systems and the three sampling time points are depicted in Additional file 3. In general, the four most represented pathways across systems were, Membrane transport (11.1% at TP1; 11.5% at TP2; 11.9% at TP3), Replication and Repair (9.8% at TP1; 10.2% at TP2; 10.0% at TP3), Carbohydrate Metabolism (9.9% at TP1; 9.8% at TP2; 9.6% at TP3), and Amino Acid Metabolism (9.3% at TP1; 9.1% at TP2; 9.1% at TP3) (Additional file 3; panel a). Pathway differences among systems are reported in Additional file 3 panel b. Membrane Transport (TP1 and TP3), Cell Motility (TP1), Transcription (TP1), Replication and Repair (TP2), Translation (TP2), Glycan Biosynthesis and Metabolism (TP2), and Energy Metabolism (TP2) were over-represented pathways in TE. In contrast, Glycan Biosynthesis and Metabolism (TP1), Carbohydrate Metabolism (TP1), Membrane Transport (TP2), Cell Motility (TP2), and Amino Acid Metabolism (TP3) were over-represented in NU. Differences in remaining pathways between systems were small (less than 1%).

### Alpha diversity

**Fig. 2:**
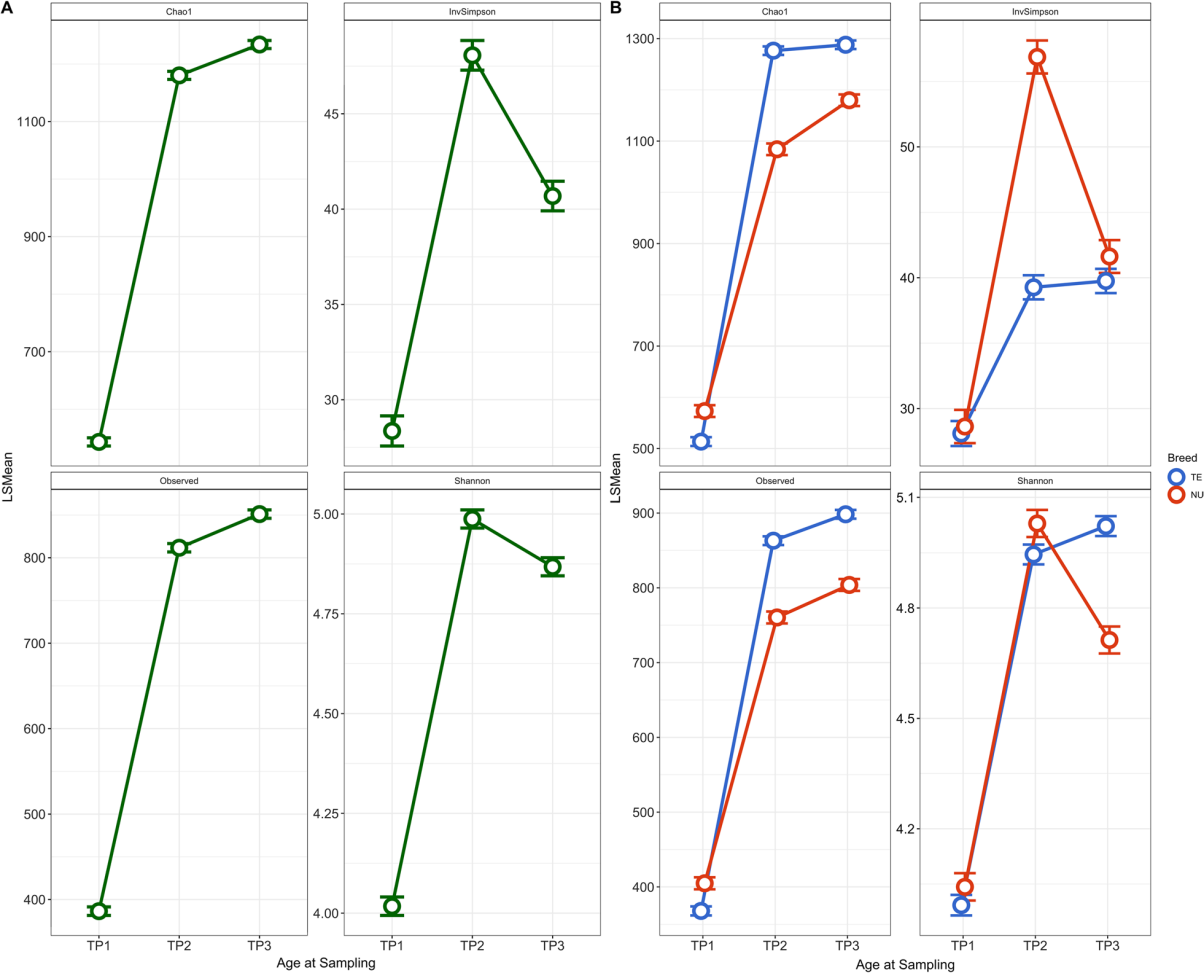
Measurements of fecal microbiome alpha diversity overall and for two systems at three time points. Measurements at OTU level using the Observed, Chao1, Shannon, Simpson, and Inverse Simpson indices (least squares means ± confidence interval) overall (**a**) and for Purebred (NU), and Crossbred (TE) (**b**) at three time points: weaning (TP1), mid test (TP2), and off test (TP3) of the feeding trial

All factors included in the model significantly affected alpha diversity with the exception of Sex and the Interaction between Sire and System, both of which were hence excluded from the final model reported in Table 1. Bacterial diversity increased with pig age according to the Observed and the Chao1 measures (Fig. 2a). The Shannon index and Inverse Simpson index, both of which weighed the evenness of taxa, increased from TP1 to TP2 and then decreased slightly (Shannon) or markedly (Inverse Shannon) at TP3 (Fig. 2a). When comparing the two populations across time, NU individuals were more diverse at TP1, regardless of the measure (Fig. 2b). At TP2 and TP3, TE individuals were more diverse according to Chao1 and Observed, while less diverse for InverseSimpson (Fig. 2b). At TP3, NU individuals were more diverse as measured by InverseSimpson, while less diverse as measured by Shannon diversity (Fig. 2b).

**Table 1:** Summary of F-value and *P*-value of factors that significantly affected alpha diversity in the model

Factor	Observed		Chao1		Shannon		InvSimpson	
	F-value	P-value	F-value	P-value	F-value	P-value	F-value	P-value
Sire	5.265	< 0.0001	4.498	< 0.0001	2.593	< 0.0001	6.793	< 0.0001
System	422.419	< 0.0001	469.364	< 0.0001	26.357	< 0.0001	199.709	< 0.0001
Time	12,800.119	< 0.0001	13,998.892	< 0.0001	2454.014	< 0.0001	600.475	< 0.0001
CG(System)	41.81	< 0.0001	39.89	< 0.0001	24.043	< 0.0001	29.945	< 0.0001
Sire:Time	4.771	< 0.0001	4.458	< 0.0001	4.17	< 0.0001	4.546	< 0.0001
Time:System	237.41	< 0.0001	314.014	< 0.0001	88.095	< 0.0001	142.133	< 0.0001

### Beta diversity and clustering

**Fig. 3:**
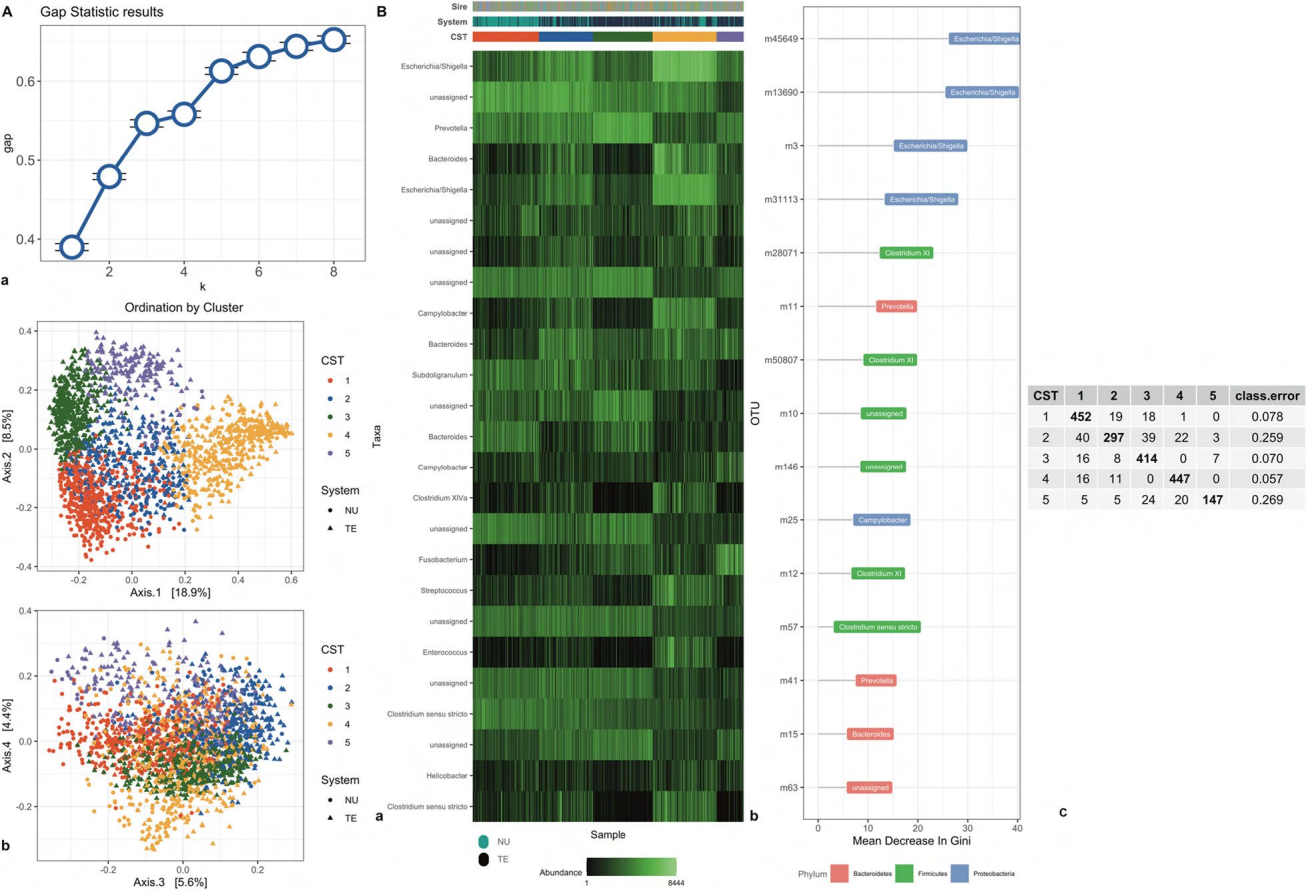

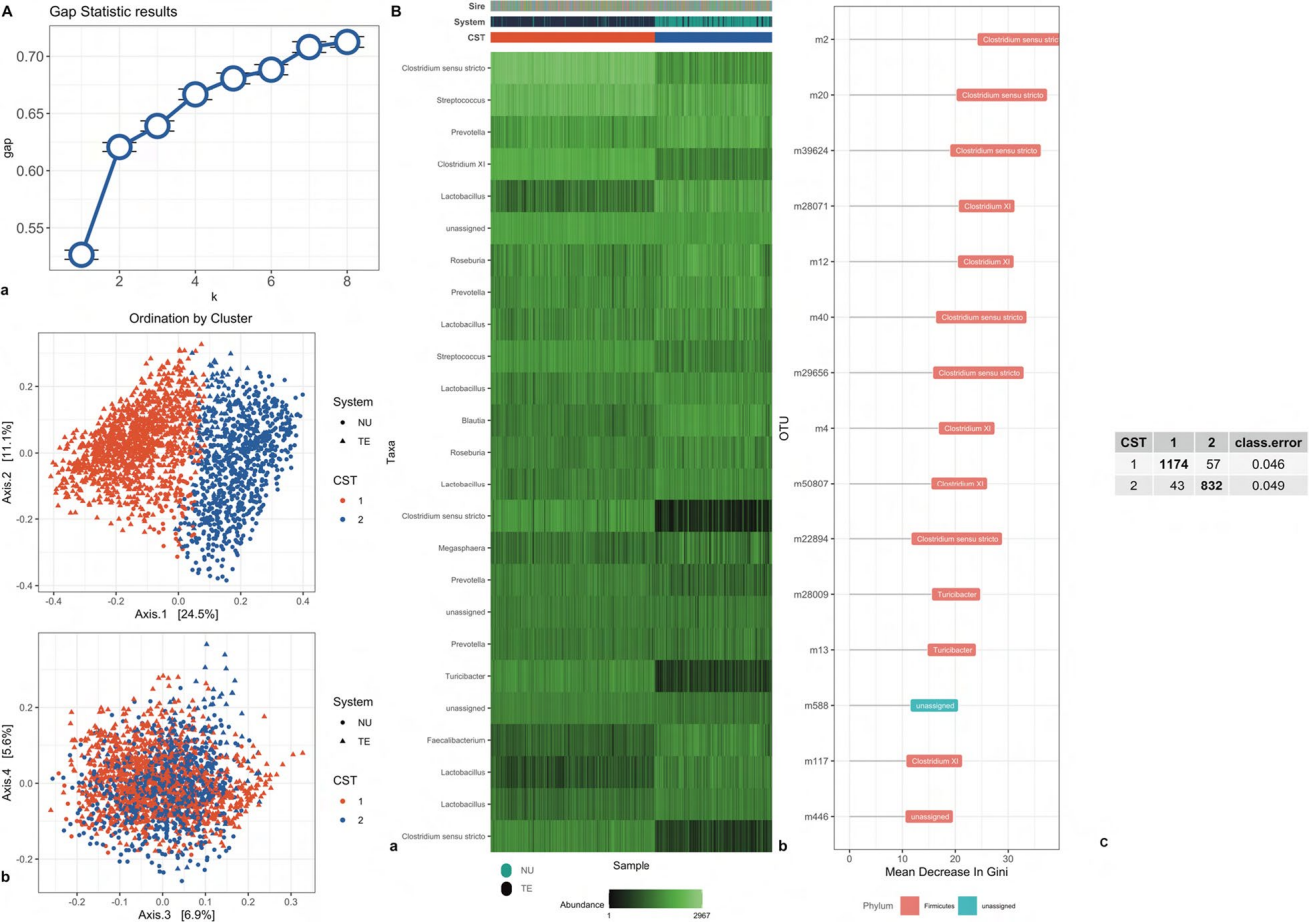

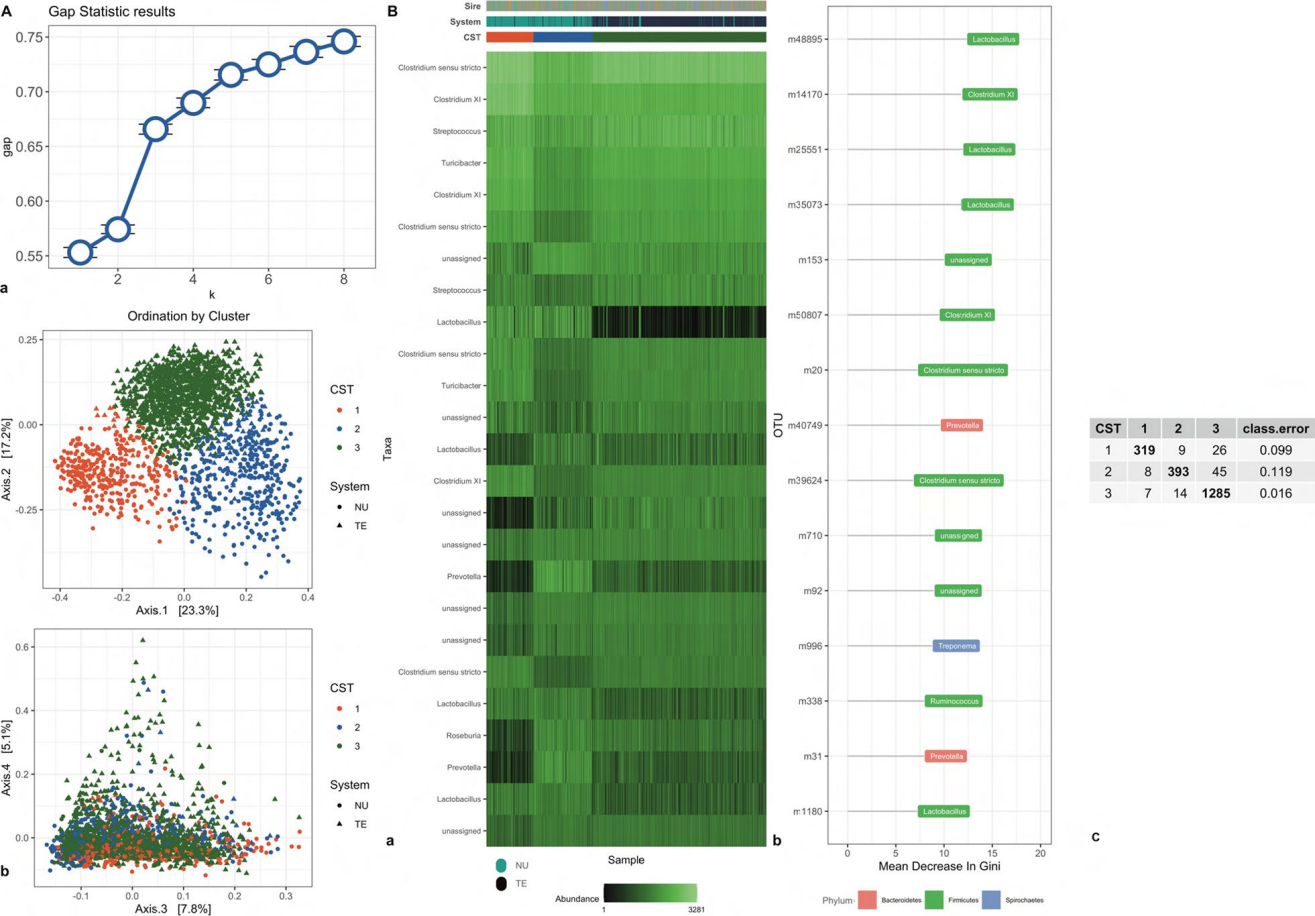
Clustering analysis of gut microbiome for two systems at three time points. Clustering analysis of gut microbiome data collected for Purebred (NU) and Crossbred (TE) (**b**) at three time points: weaning (TP1), mid test (TP2), and off test (TP3) of the feeding trial. Gap statistic (**a**, Subpanel a) and Principal Coordinates Analysis (PCoA) (**a**, Subpanel, b). Genus representation (**b**, subpanel a) and variable importance (**b**, Subpanel b). Breed (TE, NU), CST (cluster 1–5). Confusion matrix (**b**, Subpanel b). On the diagonal individuals classified in the correct cluster. Off diagonal number individuals misclassified to different clusters. The last column represents the error rate in classification

The clustering of individuals at each time of sampling and the top 15 important variables in discriminating each cluster (CST) are depicted in Fig. 3. Using the gap statistics, we identified five clusters at TP1, two at TP2, and three at TP3. At TP1, the clusters separated NU and TE individuals markedly (Fig. 3 TP1; panel A and B). Cluster one included mostly NU individuals, while cluster three included mostly TE individuals. The remaining clusters were a mixture of the two systems. At the phylum level, most of the clustering was determined by OTUs of the Proteobacteria and Firmicutes phyla. At the genus level, clusters were discriminated mostly by OTUs of the *Escherichia/Shigella* genera, which was prominent in cluster four. At TP2, clustering recapitulated the system split of the experimental design with two clusters identified, with cluster one including almost exclusively TE and cluster two NU individuals (Fig. 3 TP2; panel A and B). Firmicutes of the genus *Clostridium *sensu stricto and *Clostridium XI* were the largest cluster determinants. At TP3, three clusters were identified (Fig. 3 TP3; panel A and B). The TE individuals were almost entirely assigned to cluster three, while NU individuals were assigned to the remaining two clusters. The largest driver of cluster three was the genus *Lactobacillus*, which was more abundant in clusters one and two. Conversely, the genus *Prevotella* discriminated between clusters one and two.

**Fig. 4:**
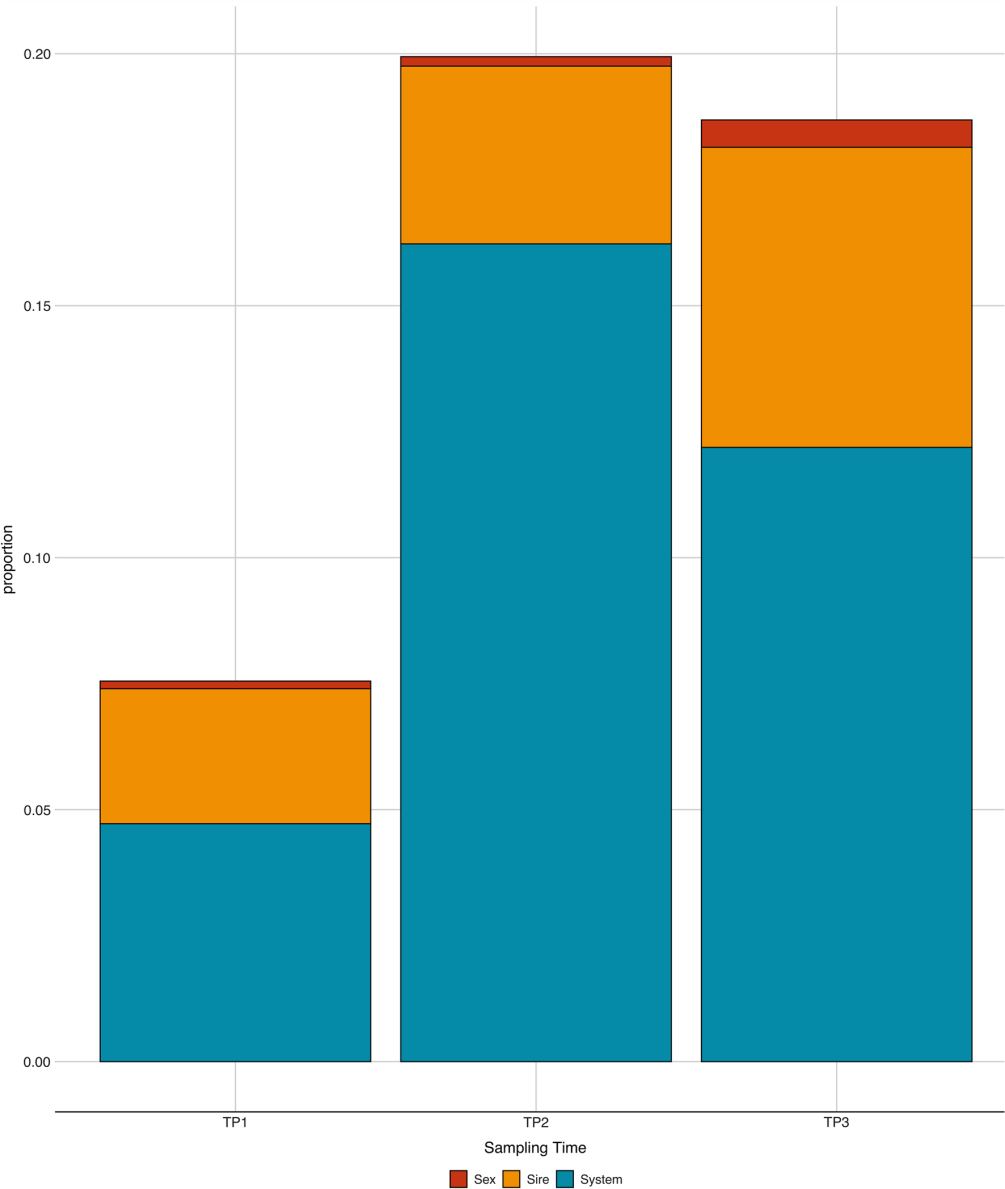
The contribution to the total R2 of each effect in the model. Permanova $R2$ contribution of each effect to the overall model for Purebred (NU) and Crossbred (TE) at three time points: weaning (TP1), mid test (TP2), and off test (TP3) of the feeding trial

In the PERMANOVA analysis, at all three time points, System and Sire were significant (adjusted *P* < 0.01, results not shown), while Sex was only significant at TP3. We reported the contribution to the total R2 of each effect in the model in Fig. 4. At TP1, the effect with the most substantial contribution was System (4.7% of R2) followed by Sire (2.7% of R2). At TP2, System had the largest R2 (16.2%), followed by Sire (3.5%). Similar trends were seen at TP3, where the contribution of Sire increased to 6% of the total R2, while System contributed 12.1%, and sex 0.05%. In general, at later samplings, cumulatively, the model’s effects explained more variance, increasing from ~ 9 to ~ 19% across time points.

### Differentially abundant microbes

**Fig. 5:**
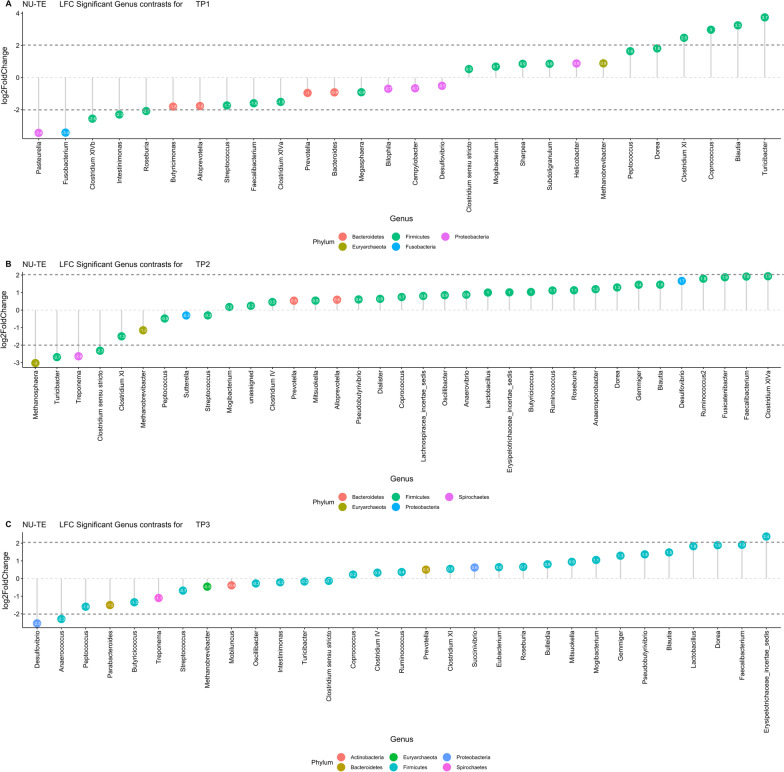
Genera differential abundance between NU and TE systems for the three sampling points. Results are expressed as Log2FoldChange (LFC) for Nucleus (NU), and Commercial (TE) at three time points: weaning (TP1 **a**), mid test (TP2 **b**), and off test (TP3 **c**) of the feeding trial

Genera differential abundance between NU and TE expressed as *Log2FoldChange* for the three sampling points is reported in Fig. 5 for genera with adjusted *P* < 0.01 (FDR). There were 16 significantly different genera with an absolute Log2FoldChange of at least one among NU and TE at TP1. Of these, 75% (12) were of phylum Firmicutes. The genera with the largest Log2FoldChange were *Pasteurella* (-3.4 in TE) and *Turicibacter* (+ 3.7 in NU), followed by *Fusobacterium* (-3.4 in TE) and *Blautia* (+ 3.3 in NU).

At TP2, there were 20 genera significantly different with an absolute Log2FoldChange of at least one among NU and TE. The most represented Phylum was again Firmicutes (16), followed by Euryarchaeota (2), Proteobacteria and Spirochaetes (1 each). *Methanosphaera Turicibacter* and *Treponema* (-3. -2.7, -2.6 in TE) and *Clostridium XlVa*, *Faecalibacterium* and *Fusicatenibacter* (+ 1.9, + 1.9, + 1.9 in NU) were the genera with largest Log2FoldChange at TP2.

Fourteen genera were significantly different at TP3, 11 of these belonged to Firmicutes phylum while the others were Bacteroidetes, Proteobacteria, and Spirochaetes. *Desulfovibrio*, *Anaerococcus* and *Peptococcus* (− 2.5, − 2.3, − 1.6 in TE), and *Erysipelotrichaceae_incertae_sedis*, *Faecalibacterium* and *Dorea* (+ 2.4, + 1.9, + 1.9 in NU) were the genera with the largest differences.

### Traits association

**Fig. 6:**
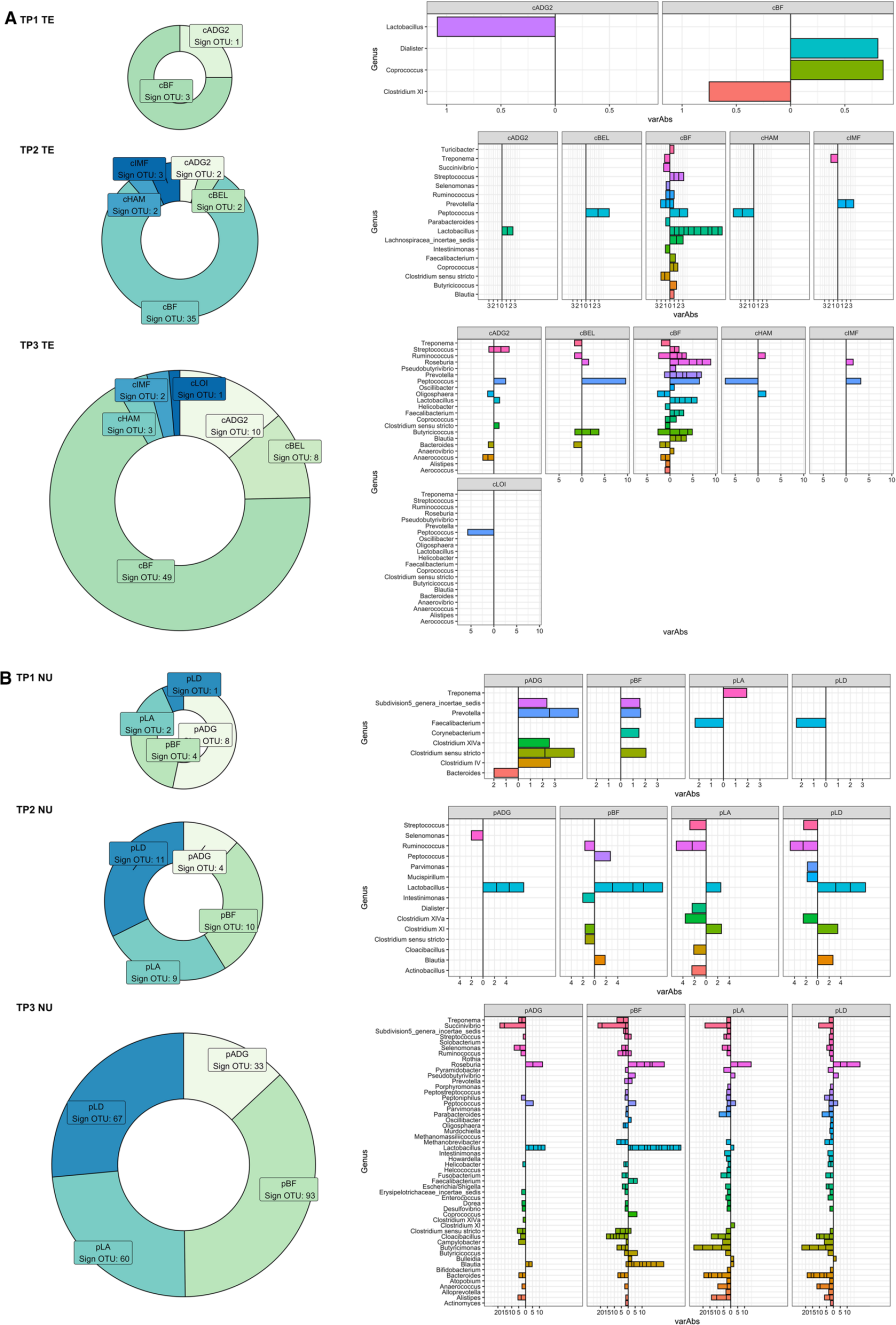
Summary of significant trait OTUs associations. Significant OTU association for different traits (Left Panel) and variance absorbed by Genus (left panel, the direction of the bar indicates the sign of the effect: left negative, right positive) for Commercial (TE, **a**), and Nucleus (NU, **b**) at three time points: weaning (TP1), mid test (TP2), and off test (TP3) of the feeding trial

We obtained trait OTUs associations for each of the two populations at each of the census points. The results are summarized in Fig. 6 and Table 2. There were 656 and 1 012 unique significant OTUs identified at an adjusted *P* < 0.05 for TE and NU. Of these 182 264, and 566 for NU; and 67 221, and 368 for TE, at TP one, two, and three, respectively. Eight of the 13 traits considered in TE had at least one OTU associated at one of the three sampling times, while all of the six traits investigated in NU had at least one OTU significantly associated.

**Table 2:** Summary of OTUs significantly associated with growth traits in two systems

Trait	Time	OTU	Genus	b TE	SE TE	Var% TE	adj-p TE	b NU	SE_NU	Varr%_NU	adj-p NU
ADG	TP2	m263	Collinsella	− 1.550	0.350	1.638	0.001	− 1.656	0.505	1.364	0.024
ADG	TP2	m560	Peptococcus	2.191	0.260	5.885	0.000	1.498	0.427	2.164	0.016
ADG	TP2	m1178	Selenomonas	− 1.657	0.258	3.300	0.000	− 1.603	0.419	2.011	0.008
ADG	TP3	m147	Peptococcus	1.676	0.353	2.095	0.000	1.834	0.285	5.494	0.000
ADG	TP3	m40684	Prevotella	− 1.075	0.345	0.832	0.041	− 1.644	0.559	2.894	0.045
ADG	TP3	m68	Prevotella	− 1.558	0.471	0.913	0.028	− 1.631	0.515	1.819	0.030
BF	TP2	m1090	Clostridium sensu stricto	− 1.017	0.365	0.523	0.048	− 1.244	0.347	1.609	0.007
BF	TP2	m370	Clostridium sensu stricto	− 0.838	0.274	0.648	0.029	− 0.974	0.344	1.346	0.039
BF	TP2	m1641	Lactobacillus	1.177	0.292	1.039	0.002	1.107	0.384	1.192	0.035
BF	TP2	m17	Lactobacillus	1.032	0.246	1.335	0.002	2.325	0.470	3.508	0.000
BF	TP2	m28987	Lactobacillus	1.048	0.244	1.416	0.001	2.171	0.461	3.298	0.000
BF	TP2	m33615	Lactobacillus	0.956	0.201	1.657	0.000	0.934	0.329	1.297	0.038
BF	TP2	m35073	Lactobacillus	0.498	0.170	0.689	0.038	1.105	0.357	1.363	0.023
BF	TP2	m52033	Lactobacillus	0.967	0.248	1.155	0.004	2.166	0.472	3.120	0.000
BF	TP2	m9	Lactobacillus	0.930	0.202	1.542	0.001	0.933	0.321	1.301	0.034
BF	TP2	m560	Peptococcus	1.337	0.236	2.201	0.000	1.686	0.410	2.738	0.002
BF	TP3	m470	Bacteroides	− 1.468	0.395	0.979	0.006	− 2.512	0.878	0.959	0.037
BF	TP3	m638	Bacteroides	− 1.322	0.462	0.530	0.042	− 4.532	1.469	1.234	0.023
BF	TP3	m16	Blautia	0.538	0.186	0.635	0.039	1.069	0.274	2.120	0.003
BF	TP3	m472	Blautia	1.286	0.308	1.170	0.002	1.661	0.367	3.266	0.000
BF	TP3	m564	Blautia	1.199	0.273	1.265	0.001	1.867	0.358	4.103	0.000
BF	TP3	m595	Blautia	1.874	0.464	1.148	0.002	2.080	0.320	5.407	0.000
BF	TP3	m649	Blautia	1.003	0.322	0.625	0.026	1.040	0.351	1.282	0.030
BF	TP3	m2023	Butyricicoccus	1.511	0.296	1.783	0.000	1.877	0.406	6.652	0.000
BF	TP3	m80	Butyricicoccus	− 1.547	0.248	2.627	0.000	− 1.277	0.332	2.651	0.004
BF	TP3	m1540	Clostridium sensu stricto	− 0.805	0.266	0.612	0.031	− 2.958	0.744	2.058	0.002
BF	TP3	m40	Clostridium sensu stricto	− 1.908	0.602	0.887	0.023	− 2.175	0.615	3.745	0.008
BF	TP3	m57	Clostridium sensu stricto	− 0.687	0.179	1.013	0.004	− 1.616	0.558	0.960	0.034
BF	TP3	m518	Clostridium XlVa	1.599	0.540	0.599	0.035	1.336	0.473	1.224	0.039
BF	TP3	m88	Coprococcus	0.868	0.203	1.369	0.001	1.712	0.387	6.359	0.001
BF	TP3	m69	Dorea	− 2.756	0.866	0.693	0.023	− 3.235	0.827	2.053	0.003
BF	TP3	m23	Faecalibacterium	0.735	0.201	0.998	0.007	1.237	0.290	3.830	0.001
BF	TP3	m27249	Faecalibacterium	0.917	0.237	1.078	0.004	0.886	0.309	1.707	0.036
BF	TP3	m37847	Faecalibacterium	0.754	0.237	0.746	0.023	1.121	0.321	2.759	0.009
BF	TP3	m2495	Helicobacter	− 1.142	0.287	1.017	0.003	− 2.405	0.679	1.484	0.008
BF	TP3	m561	Helicobacter	− 1.099	0.334	0.692	0.017	− 3.036	0.795	1.683	0.004
BF	TP3	m1834	Lachnospiracea_incertae_sedis	0.881	0.315	0.494	0.047	− 1.766	0.517	1.285	0.011
BF	TP3	m17	Lactobacillus	0.584	0.197	0.613	0.035	2.130	0.381	4.050	0.000
BF	TP3	m28987	Lactobacillus	0.531	0.187	0.567	0.043	1.933	0.346	3.953	0.000
BF	TP3	m327	Methanobrevibacter	− 0.931	0.277	0.786	0.015	− 1.226	0.330	2.232	0.005
BF	TP3	m478	Murdochiella	− 0.579	0.198	0.594	0.037	− 1.397	0.406	1.590	0.010
BF	TP3	m10347	Oligosphaera	− 1.089	0.251	1.210	0.001	− 2.363	0.560	2.216	0.001
BF	TP3	m851	Oligosphaera	− 1.514	0.313	1.499	0.000	− 3.752	1.084	1.384	0.009
BF	TP3	m796	Oscillibacter	1.466	0.389	0.939	0.005	1.888	0.656	1.339	0.035
BF	TP3	m147	Peptococcus	2.935	0.304	6.434	0.000	1.840	0.274	5.505	0.000
BF	TP3	m477	Peptococcus	− 0.608	0.212	0.560	0.041	− 0.932	0.344	1.043	0.049
BF	TP3	m272	Peptoniphilus	− 0.639	0.193	0.734	0.016	− 1.199	0.317	2.257	0.004
BF	TP3	m68	Prevotella	− 1.315	0.419	0.651	0.025	− 1.947	0.492	2.574	0.003
BF	TP3	m326	Pseudobutyrivibrio	0.866	0.300	0.557	0.040	1.964	0.369	5.067	0.000
BF	TP3	m19	Roseburia	0.981	0.204	1.656	0.000	1.134	0.311	4.060	0.006
BF	TP3	m255	Roseburia	1.249	0.241	1.846	0.000	1.833	0.389	8.778	0.000
BF	TP3	m294	Roseburia	1.257	0.213	2.373	0.000	1.859	0.354	5.378	0.000
BF	TP3	m325	Roseburia	1.451	0.279	1.838	0.000	1.792	0.395	6.258	0.000
BF	TP3	m628	Roseburia	1.423	0.331	1.200	0.001	1.327	0.387	2.404	0.010
BF	TP3	m955	Roseburia	1.409	0.479	0.552	0.037	1.757	0.501	1.610	0.009
BF	TP3	m688	Ruminococcus	− 1.568	0.261	2.470	0.000	− 2.876	0.519	3.558	0.000
BF	TP3	m122	Streptococcus	− 0.739	0.253	0.577	0.038	− 2.169	0.459	2.474	0.000
BF	TP3	m910	Streptococcus	1.082	0.285	0.993	0.005	1.374	0.363	2.188	0.004
BF	TP3	m1571	Subdivision5_genera_incertae_sedis	− 0.885	0.311	0.523	0.043	− 1.549	0.543	0.938	0.037
BF	TP3	m557	Subdivision5_genera_incertae_sedis	− 1.537	0.466	0.712	0.017	− 1.203	0.378	1.348	0.019
BF	TP3	m878	Subdivision5_genera_incertae_sedis	− 1.070	0.327	0.712	0.018	− 1.233	0.402	1.784	0.024
BF	TP3	m53	Succinivibrio	− 0.687	0.228	0.632	0.032	− 2.642	0.439	16.999	0.000
BF	TP3	m801	Succinivibrio	− 1.139	0.333	0.832	0.013	− 2.035	0.582	1.883	0.009
BF	TP3	m224	Treponema	− 1.218	0.236	1.878	0.000	− 3.836	0.591	4.715	0.000
IMF	TP3	m147	Peptococcus	2.070	0.345	3.205	0.000	0.901	0.215	1.318	0.022

Figure 6a reports the association results for OTUs with an adjusted *P* < 0.01 on the TE population. On the left panel, the number of significant OTU is depicted. The magnitude of variance explained by each OTU in the model is instead reported on the right panel, with the direction of the bar indicating whether the effect of the OTU was positive or negative. At TP1, only four OTUs were significantly associated with phenotypic performance, three for BF, and one for ADG. At TP2, a total of 45 OTUs were associated with performance traits. The largest proportion, ~ 77%, was associated with BF. Significant OTUs were mostly from two genera, *Lactobacillus* and *Peptococcus*. For the OTUs of both genera, an increase in abundance was associated with an increase in BF. Conversely, some of the *Peptococcus* OTUs were associated with a decrease in ham yield. Similarly, at TP3, a large part of the associations was with BF (49 of the 73 significant associations), followed by ADG and BEL. The direction of the effect was consistent, yet the magnitude was larger, as shown by the variance explained. Interestingly while most of the growth traits were associated with several OTUs, few associations were identified for carcass quality and composition.

Similar general trends were observed in NU (Fig. 6b), with the number of significant associations increasing from 15 at TP1 to 34 at TP2. At TP3, the number of significant associations increased significantly, with 253 total associations identified. Again the most substantial proportion was for BF (37%), followed by LA, LD, and ADG. Interestingly, at TP3, a more diverse group of genera was represented. Members of the genus *Succinivibrio* negatively impacted all traits, while OTUs of the genus *Roseburia* had a positive association. The magnitude of the variance absorbed was sizable, ranging from 5% to almost 20%. *Lactobacillus* and *Peptococcus* OTUs showed a similar magnitude and direction in NU than in TE for fat deposition and daily gain. OTUs of the *Blautia* genus were positively associated with daily gain and fat deposition. In contrast, an increase in the presence of members of the *Bacteroides* genus was negatively associated with growth performance parameters. A complete list of results for trait associations is reported in Additional file 4.

Most of the growth traits were consistently recorded across the two systems. For these traits (ADG, BF, LD, and IMF), the number of OTUs significantly associated (adjusted *P* < 0.05) with each trait in both populations is reported in Table 2. There were 14 OTUs that were significant in both populations for ADG, with one at TP2 and 13 at TP3. Of these, the largest number (6) belonged to the genus *Lactobacillus*. For BF, there were 10 OTUs in common between TE and NU at TP2. Seven of the genus *Lactobacillus*, two of the genus *Clostridium *sensu stricto, and one of the genus *Peptococcus*. At TP3, 16 OTUs were in common, seven of genus *Lactobacillus*, five belonging to the genus *Blautia*, and three of genus *Clostridium *sensu stricto. A single *Peptococcus* OTU was significant in both populations at TP3 for IMF, while none were found for LD.

## Discussion

In this paper, we investigated the impact of different production systems (Nucleus vs. Commercial) on microbiome composition in swine. Subsequently, we identified microbial OTUs associated with carcass composition in each of the two systems and in common among the two. To the best of our knowledge, this is one of the few and probably the largest study in this regard. Following, we highlight a few key points on the experimental design and analysis.

The current study expands a trial we previously conducted on the TE population. As such, partial non-redundant results of the present research on TE have been published earlier. We have focused our previous studies on the inclusion of microbial information in predictive models for selection purposes through microbial covariance matrices [48]. Here, we significantly extend these results by providing a comprehensive ecological comparison of nucleus versus terminal systems, meanwhile essentially doubling the sample size of the analysis. Furthermore, we present the association of microbial profiles with carcass quality parameters for both the NU and the TE, which has not been shown before. Within this research, we ran the bioinformatics pipeline de novo on the entire dataset (thus including both TE and NU). To maintain a connection with the previously published work, we decided to keep the processing of sequence information as close as possible to our previous analyses. This meant utilizing OTUs as opposed to ASV and the use of the Greengenes database as opposed to Silva [51] for taxonomic classification. While we recognize some of the disadvantages of our choice, we believe that the ability to compare results from the current study with previous work from our and other groups outweighs the drawbacks.

Microbial composition varied between TE and NU, with differences in abundance more marked at TP1. At TP3, the two populations were similar at the phylum level, with the most substantial contribution to the overall communities of Firmicutes and Bacteroidetes, which is consistent with literature results [52]. In contrast, at the genus level, the two populations were more different. Previous research [23, 53, 54] has shown how different breeds of pigs have distinct microbial profiles. In this research, differences in composition were less prominent, probably reflecting all individuals’ common origin from the 28 founding sires. Alpha diversity over time followed a typical swine pattern [55, 56], with an overall increase in diversity from TP1 to TP3. Differences among breeds over time were identified by Bergamaschi et al. [23] using Duroc, Large White, and Landrace populations. In our study, NU included purebred individuals from the Duroc breed, while TE included crossbred individual crosses between the Duroc sires and F1 crossbred dams. For the most part, results from our data recapitulate those of their study, with NU having lower diversity at TP1 increasing significantly at TP2 and with a sharper decline at TP3 compared to TE.

Pathways abundance was dominated by carbohydrate, amino acid, energy, and lipid metabolism across populations and time, along with membrane transport and replication and repair. These results are again in agreement with previous literature [57, 58]. When comparing the two populations, glycan amino acid and energy metabolism were less abundant in TE than NU at time points one and three, while the opposite was true at TP2. It has been shown before that growth patterns of the nucleus and terminal lines differ [50], and genetic correlations of growth and carcass composition in the two systems are less than unity [59]. These differences could, at least in part, be attributed to a different evolution of the microbial communities in the two different populations.

We performed a cluster analysis to identify core OTUs separating individuals at different time points. We found that for the most part the clustering recapitulated the system separation and that clustering was not consistent over time. Our results differ from other studies [60, 61], which identified stable *Prevotella* and *Ruminococcus* enterotypes. Within this study the cluster was collinear with the system, although, at TP1, not entirely. Bacteria of the *Escherichia-Shigella* genus were the largest cluster discriminates at TP1. Bacteria of this genus are facultative anaerobe and include several opportunistic pathogens. Bin et al. [62] showed how diarrheal piglets have an increased percentage of *Escherichia* in feces, possibly highlighting a different health status of different individuals close to sampling time at TP1 in our study. In addition, Guevarra and colleagues [20, 63] showed how the fecal microbiome of the nursing piglets has a higher abundance of *Bacteroides* bacteria, a group enriched in the utilization of lactose and galactose. On the other hand, in the same study, *Prevotella* and *Lactobacillus* associated with carbohydrate and amino acid metabolism, were enriched after weaning. Some of the same genera were also discriminating clusters at TP1. Weaning is a transition period for the piglet, which coincides with a drastic switch of the diet away from the maternal milk. It is possible that some of the differences identified in this study in the clustering of individuals at TP1 are related to the ability of each piglet to adapt more or less quickly to the new diet, regardless of the system. Tools that use microbial information to classify and identify individuals that are transitioning to the new diet faster or are at less than favorable health status could be used in either (re)grouping individuals at weaning, or through additional supplementation or dietary remediation treatments. Furthermore, the possibility of identifying a proportion of individuals classified as challenged based on microbial information, could be used as a tool to benchmark the environmental and management status of a farm as compared to either a baseline or other farms in similar systems. Results from our current work show how clusters might capture systematic variability not captured by genetics or other systematic background effects, but further research would be needed in this regard. Previous research reported a significant effect of the host genomic makeup in shaping the gut microbial population of swine [14, 24]. In our study, Sire was significant in shaping microbial community regardless of the system, confirming some of these previous results. At TP3, the two systems were separated markedly by bacteria of the genus *Lactobacillus*, with a higher prevalence in the NU system. This group of bacteria are characterized by the production lactic acid as the metabolic end-product of carbohydrate fermentation. *Lactobacillus* are widely used as probiotic to improve growth performance, feed conversion efficiency and nutrient utilization. The lower abundance in the TE system might have several explanations. Lower concentration of *Lactobacillus* might reflect a more challenging environment of individuals in the commercial facilities. *Lactobacillus* are modulators immune system in pigs and their abundance might reflect higher levels of general stress consequence of a less controlled environment at the TE level. Additionally, we have previously reported that taxa of the *Lactobacillus* genus are heritable [24], and this difference might reflect the genetic makeup of the crossbred vs. purebred individuals. Further research would be nonetheless needed to confirm results of the current study. Within the NU system, two groups were identified mainly separated by bacteria of genus *Roseburia* and *Prevotella*. Recent literature has associated members of the genus *Prevotella* with positive outcomes in pig production, including growth performance [64] and immune response [65]. Within the NU system the ability different microbial compositions related to altered performance could be used in the context of selection. For instance, abundance of significantly discriminant taxa could be used to better adjust performance of individuals (similarly to other systematic effects, such as for example pen or batch) thus allowing a better discrimination of the true genetic potential of individuals, resulting in higher accuracy breeding values and increased selection efficiency.

Specifically, when comparing differential genus abundance over time between NU and TE at TP1, the largest differences were identified for *Pasteurella*, *Fusobacterium* and *Coprococcus*. At TP2 *Methanosphaera* was the genus with the largest logfold change across systems. A study of Luo [66] linked a higher diversity of this genus to leaner breeds of pigs. In our study in the nucleus individuals were purebred Durocs while in the commercial system were terminal crossing, possibly suggesting a host role in this difference. Differential abundance of several genera has been presented in pigs in association with changes in diet management conditions or growth efficiency [57, 67]. In our study, differences were more marked at weaning, while later differences were of lesser magnitude.

The association of microbial OTUs with carcass and quality traits highlighted how different OTUs were associated in the two different populations but with some core genera in common. In both populations, fewer associations were identified at TP1, while an increasing number was identified at time points two and three, consistently with results from [23]. In NU, the OTUs of the genus *Lactobacillus* were associated with an increase in both growth rate as well as fatness both at time points two and three. Several species of the genus *Lactobacillus* have been linked to performance in swine [68]. Lactobacilli improve swine energy metabolism, participating both in the maintenance of the integrity of the intestinal tract and modulating the immune responses in swine [69]. Recently *Lactobacillus* spp., have been linked to a suppression of swine feed intake [70] and with feed efficiency [71]. Additionally, in NU we identified an association between OTUs of the genus *Roseburia*, and growth parameters which were previously reported by Bergamaschi et al. [23] when comparing the Duroc breed with Landrace and Large white, and by Tan and colleagues [72] in association with differences in feed efficiency among pigs.

*Peptococcus* spp. were significantly associated with fat deposition and growth at time points two and three in TE as previously published by [21]. The association between *Peptococcus* bacteria and BF and ADG was also identified in NU, although the variance explained in this case was smaller. A recent paper by Oh and colleagues [73] found similar associations between *Peptococcus* spp., body weight, and average daily gain in growing pigs.

The OTUs of the genera *Lactobacillus*, *Blautia*, *Peptococcus*, and *Clostridium* represented the vast majority of the significant association in common across the two populations. Several of these were identified as part of the core gut microbiota by Holman et al., [52]. The direction of the average correlation between effects among the common OTUs was high (~ 0.88). In all cases, the direction of the effect was the same for the two populations. The average correlation between variance explained was low (~ 21%). This last result could be due to possible interactions between the genetic background and microbial communities. Similar results have been reported for genetic correlations across Nucleus and Terminal systems [74].

Several pathways were associated with growth and carcass composition in the two populations (Additional file 3). Again most of these were pathways related to energy amino acid and carbohydrates metabolism, consistently with previous research [72].

## Conclusions

Within this paper, we compared the microbial composition of two production systems that are representative of the majority of pork production organizations in North America. Differences between the nucleus and commercial backgrounds play a crucial role in determining pork production’s efficiency and profitability and are, for the most part, overlooked. We believe that this is the first attempt at characterizing such differences from the microbial communities’ perspective. We did this to understand the overall ecology of the two setups and gain a sense of how remediation/manipulation interventions to influence microbial communities developed within the nucleus system could be transferred to a commercial setting. Additionally, we aimed at collecting preliminary evidence of the possibility that lower than unity genetic correlations among production systems could be at least partially attributable to a different microbial composition. While the design of this research allowed us to control some of the intrinsic variability related to the two systems (e.g., diet and genetic background, the two major production efficiency drivers in pork production), it should be noted that other source of variation, such for example facilities layouts as well as climatic and geographical differences could not be effectively controlled within the current work. In this, further research is warranted. In the present paper, we identified both differences and similarities between the two populations investigated. While at weaning, we could not separate individuals from the two systems; as time passed, the two settings developed distinct communities, mostly differing in the *Lactobacillus* spp. abundance. Conversely, when linking OTU abundance to growth and carcass composition, we identified a common set of consistent associations in directions and a lesser extent in magnitude across the nucleus and terminal cross populations. The genus Lactobacillus, despite the different representations in the two systems, was significantly associated with fat deposition in both systems. This suggests some portability of information from one system to another, with consequent opportunities for manipulating gut microbiota that could be effective in both systems. We have, in previous work, shown how microbial composition is under partial genetic control from the host. Selecting individuals for taxa that have a positive effect on production both at the nucleus as well as the terminal level, could enhance the selection gain achievable while increasing genetic correlations between NU and TE populations. Conversely microbial information, which differs in significance among systems, could be used effectively for the same purposes as a way to control environmental variation in modeling the genetic values of individuals across systems, thus reducing the re-raking of genetic values of selected parents in commercial settings. Remediation interventions developed in nucleus populations could potentially be employed to modify microbial populations in the terminal systems. This could allow significant investment savings and provide a solution applicable to a broader spectrum of conditions. Also, remediations could curtail the effects of GxE in selection schemes in swine and shrink some differences in performance that occur between the two systems. To this extent, it should be noted that in all analyses, the genetic background of the pigs (modeled through the sire founding effect) was a significant component in shaping the microbial communities across production settings. This could be potentially exploited in breeding schemes by selecting individuals capable of maintaining a favorable microbial composition across production systems. This could serve as a potential refinement of the measures employed currently to increase energy efficiency in selected lines. Identifying cost-effective biomarkers of performance and an optimal strategy to integrate them in genetic selection schemes effectively is a priority for the US swine. Currently, little is known about how genomic selection, gut microbiome, environment, and their interaction can be used to enhance swine performance. Swine performance is a complex trait determined by factors that reside in the host genome as well as the gut microbiome. Results from our current research show that the gut microbiome is a bridging component between the host genome and the environment. Gut microbiome is an “information dense” measure and can serve as a biomarker, a predictor, or an indicator of environmental conditions. The current study provides a first characterization of microbial communities’ importance throughout the entire pork production background. Further studies should focus on further characterizing these systems and how to explicitly incorporate microbial composition into the selection process in the quest for affordable and sustainable protein production in swine.

## Supplementary Information

**Additional file 1:** Summary of diets and nutritional values provided for pigs. The table summarizes the details of diets and their nutritional values that were provided for animals involved in this study.

**Additional file 2:** Summary of vaccination and medication routine for pigs. These three tables provide information of vaccination, injectable medications, and water medications routine for animals involved in this study.

**Additional file 3:** The relative abundance of different metabolic pathways for the two systems at three time points. The figure depicts the relative abundance of metabolic pathways for Purebred (NU) and Crossbred (TE) at three time points: weaning (TP1), mid test (TP2), and off test (TP3) of the feeding trial.

**Additional file 4:** Complete results for the trait OTUs associations. The table summarizes the complete statistical results of association study between study between traits and OTUs with taxonomy annotation.

## Data Availability

The datasets supporting the conclusions of this study are available from MATATU Inc. and The Maschhoffs LLC. but restrictions apply to the availability of these data, which were used under license for the current study, and so are not publicly available. Data are however available from the authors upon reasonable request and with permission of MATATU Inc. (microbial information) and The Maschhoffs LLC (phenotypic information).

## Abbreviations

NU: Nucleus purebred population
TE: Terminal commercial crossbred population
TP1: Time point one—weaning
TP2: Time point two—mid-test
TP3: Time point three—off-test
PERMANOVA: Permutational multivariate analysis of variance
cADG: Carcass average daily gain
cLD: Loin depth
cBF: Back-fat depth
cHAM: Ham yield
cLOI: Loin yield
cYEL: Belly yield
cSCOL: Subjective measures of color
cSFIR: Subjective measures of firmness
cSMAR: Subjective measures of marbling
cMinL: Objective measures of color
cMinA: Objective measures of color
cMinB: Objective measures of color
cIMF: Intra-muscular fat deposition
cSSF: Firmness
cPH: Muscle pH
pBW: Body weight
pLD: Loin muscle depth
pLA: Loin muscle area
pBF: Back-fat depth
pIMF: Loin intra-muscular fat concentration
pADG: Average body weight daily gain
