# Supplementary material for: Microbial composition differs between production systems and is associated with growth performance and carcass quality in pigs
Source: Anim Microbiome. 2021 Aug 28;3:57. doi: 10.1186/s42523-021-00118-z (PMC8403435; doi:10.1186/s42523-021-00118-z)
Supplement: Supplementary file 4 — Additional file 4: Complete results for the trait OTUs associations. The table summarizes the complete statistical results of association study between study between traits and OTUs with taxonomy annotation. [file 42523_2021_118_MOESM4_ESM.docx]

| Trait | stage | OTU | Kingdom | Phylum | Class | Order | Family | Genus | N | Estimate | SE | varAbs | pval | fdrpval | bonpval | System |
| --- | --- | --- | --- | --- | --- | --- | --- | --- | --- | --- | --- | --- | --- | --- | --- | --- |
| pADG | TP1 | m2 | Bacteria | Firmicutes | Clostridia | Clostridiales | Clostridiaceae 1 | Clostridium sensu stricto | 780 | 2.091 | 0.531 | 2.188 | 0 | 0.006 | 0.375 | NU |
| pADG | TP1 | m233 | Bacteria | Bacteroidetes | Bacteroidia | Bacteroidales | Prevotellaceae | Prevotella | 780 | 1.684 | 0.392 | 2.568 | 0 | 0.003 | 0.077 | NU |
| pADG | TP1 | m296 | Bacteria | Firmicutes | Clostridia | Clostridiales | Ruminococcaceae | Clostridium IV | 780 | 2.099 | 0.474 | 2.64 | 0 | 0.002 | 0.043 | NU |
| pADG | TP1 | m39624 | Bacteria | Firmicutes | Clostridia | Clostridiales | Clostridiaceae 1 | Clostridium sensu stricto | 780 | 0.963 | 0.249 | 2.397 | 0 | 0.007 | 0.503 | NU |
| pADG | TP1 | m64 | Bacteria | Bacteroidetes | Bacteroidia | Bacteroidales | Prevotellaceae | Prevotella | 780 | 1.299 | 0.318 | 2.356 | 0 | 0.004 | 0.2 | NU |
| pADG | TP1 | m674 | Bacteria | Verrucomicrobia | Subdivision5 | Subdivision5_genera_incertae_sedis | Subdivision5_genera_incertae_sedis | Subdivision5_genera_incertae_sedis | 780 | 3.107 | 0.721 | 2.343 | 0 | 0.003 | 0.075 | NU |
| pADG | TP1 | m76 | Bacteria | Bacteroidetes | Bacteroidia | Bacteroidales | Bacteroidaceae | Bacteroides | 780 | -1.246 | 0.332 | 1.986 | 0 | 0.009 | 0.775 | NU |
| pADG | TP1 | m99 | Bacteria | Firmicutes | Clostridia | Clostridiales | Lachnospiraceae | Clostridium XlVa | 780 | 1.859 | 0.445 | 2.55 | 0 | 0.003 | 0.135 | NU |
| pBF | TP1 | m1997 | Bacteria | Actinobacteria | Actinobacteria | Actinomycetales | Corynebacteriaceae | Corynebacterium | 790 | 2.121 | 0.593 | 1.486 | 0 | 0.007 | 1 | NU |
| pBF | TP1 | m39624 | Bacteria | Firmicutes | Clostridia | Clostridiales | Clostridiaceae 1 | Clostridium sensu stricto | 790 | 0.896 | 0.242 | 2.065 | 0 | 0.006 | 0.969 | NU |
| pBF | TP1 | m64 | Bacteria | Bacteroidetes | Bacteroidia | Bacteroidales | Prevotellaceae | Prevotella | 790 | 1.082 | 0.31 | 1.623 | 0 | 0.009 | 1 | NU |
| pBF | TP1 | m674 | Bacteria | Verrucomicrobia | Subdivision5 | Subdivision5_genera_incertae_sedis | Subdivision5_genera_incertae_sedis | Subdivision5_genera_incertae_sedis | 790 | 2.551 | 0.703 | 1.561 | 0 | 0.007 | 1 | NU |
| pLA | TP1 | m1095 | Bacteria | Spirochaetes | Spirochaetia | Spirochaetales | Spirochaetaceae | Treponema | 790 | 1.93 | 0.535 | 1.914 | 0 | 0.01 | 1 | NU |
| pLA | TP1 | m440 | Bacteria | Firmicutes | Clostridia | Clostridiales | Ruminococcaceae | Faecalibacterium | 790 | -2.241 | 0.581 | 2.317 | 0 | 0.005 | 0.517 | NU |
| pLD | TP1 | m440 | Bacteria | Firmicutes | Clostridia | Clostridiales | Ruminococcaceae | Faecalibacterium | 790 | -2.289 | 0.56 | 2.418 | 0 | 0.002 | 0.195 | NU |
| pADG | TP2 | m1178 | Bacteria | Firmicutes | Negativicutes | Selenomonadales | Veillonellaceae | Selenomonas | 786 | -1.603 | 0.419 | 2.011 | 0 | 0.008 | 0.594 | NU |
| pADG | TP2 | m17 | Bacteria | Firmicutes | Bacilli | Lactobacillales | Lactobacillaceae | Lactobacillus | 786 | 1.836 | 0.491 | 2.189 | 0 | 0.01 | 0.821 | NU |
| pADG | TP2 | m52033 | Bacteria | Firmicutes | Bacilli | Lactobacillales | Lactobacillaceae | Lactobacillus | 786 | 1.875 | 0.491 | 2.34 | 0 | 0.008 | 0.61 | NU |
| pADG | TP2 | m9 | Bacteria | Firmicutes | Bacilli | Lactobacillales | Lactobacillaceae | Lactobacillus | 786 | 1.291 | 0.33 | 2.496 | 0 | 0.006 | 0.417 | NU |
| pBF | TP2 | m1090 | Bacteria | Firmicutes | Clostridia | Clostridiales | Clostridiaceae 1 | Clostridium sensu stricto | 796 | -1.244 | 0.347 | 1.609 | 0 | 0.007 | 1 | NU |
| pBF | TP2 | m117 | Bacteria | Firmicutes | Clostridia | Clostridiales | Peptostreptococcaceae | Clostridium XI | 796 | -1.381 | 0.393 | 1.623 | 0 | 0.008 | 1 | NU |
| pBF | TP2 | m17 | Bacteria | Firmicutes | Bacilli | Lactobacillales | Lactobacillaceae | Lactobacillus | 796 | 2.325 | 0.47 | 3.508 | 0 | 0 | 0.003 | NU |
| pBF | TP2 | m252 | Bacteria | Firmicutes | Clostridia | Clostridiales | Lachnospiraceae | Blautia | 796 | 1.698 | 0.444 | 1.824 | 0 | 0.004 | 0.597 | NU |
| pBF | TP2 | m2559 | Bacteria | Firmicutes | Bacilli | Lactobacillales | Lactobacillaceae | Lactobacillus | 796 | 1.308 | 0.357 | 1.811 | 0 | 0.006 | 1 | NU |
| pBF | TP2 | m28987 | Bacteria | Firmicutes | Bacilli | Lactobacillales | Lactobacillaceae | Lactobacillus | 796 | 2.171 | 0.461 | 3.298 | 0 | 0 | 0.011 | NU |
| pBF | TP2 | m463 | Bacteria | Firmicutes | Clostridia | Clostridiales | Ruminococcaceae | Intestinimonas | 796 | -0.929 | 0.259 | 2.027 | 0 | 0.007 | 1 | NU |
| pBF | TP2 | m52033 | Bacteria | Firmicutes | Bacilli | Lactobacillales | Lactobacillaceae | Lactobacillus | 796 | 2.166 | 0.472 | 3.12 | 0 | 0 | 0.02 | NU |
| pBF | TP2 | m560 | Bacteria | Firmicutes | Clostridia | Clostridiales | Peptococcaceae 1 | Peptococcus | 796 | 1.686 | 0.41 | 2.738 | 0 | 0.001 | 0.176 | NU |
| pBF | TP2 | m5720 | Bacteria | Firmicutes | Clostridia | Clostridiales | Ruminococcaceae | Ruminococcus | 796 | -1.489 | 0.415 | 1.671 | 0 | 0.007 | 1 | NU |
| pLA | TP2 | m1015 | Bacteria | Proteobacteria | Gammaproteobacteria | Pasteurellales | Pasteurellaceae | Actinobacillus | 796 | -12.009 | 2.927 | 2.45 | 0 | 0.002 | 0.183 | NU |
| pLA | TP2 | m122 | Bacteria | Firmicutes | Bacilli | Lactobacillales | Streptococcaceae | Streptococcus | 796 | -3.686 | 0.846 | 2.807 | 0 | 0.001 | 0.059 | NU |
| pLA | TP2 | m2570 | Bacteria | Synergistetes | Synergistia | Synergistales | Synergistaceae | Cloacibacillus | 796 | -12.669 | 3.317 | 2.121 | 0 | 0.005 | 0.603 | NU |
| pLA | TP2 | m468 | Bacteria | Firmicutes | Negativicutes | Selenomonadales | Veillonellaceae | Dialister | 796 | -5.246 | 1.295 | 2.4 | 0 | 0.002 | 0.23 | NU |
| pLA | TP2 | m52033 | Bacteria | Firmicutes | Bacilli | Lactobacillales | Lactobacillaceae | Lactobacillus | 796 | 1.953 | 0.53 | 2.539 | 0 | 0.008 | 1 | NU |
| pLA | TP2 | m5720 | Bacteria | Firmicutes | Clostridia | Clostridiales | Ruminococcaceae | Ruminococcus | 796 | -1.782 | 0.462 | 2.393 | 0 | 0.005 | 0.511 | NU |
| pLA | TP2 | m688 | Bacteria | Firmicutes | Clostridia | Clostridiales | Ruminococcaceae | Ruminococcus | 796 | -2.992 | 0.694 | 2.746 | 0 | 0.001 | 0.074 | NU |
| pLA | TP2 | m868 | Bacteria | Firmicutes | Clostridia | Clostridiales | Lachnospiraceae | Clostridium XlVa | 796 | -6.665 | 1.327 | 3.588 | 0 | 0 | 0.002 | NU |
| pLA | TP2 | m89 | Bacteria | Firmicutes | Clostridia | Clostridiales | Peptostreptococcaceae | Clostridium XI | 796 | 1.837 | 0.47 | 2.655 | 0 | 0.004 | 0.424 | NU |
| pLD | TP2 | m122 | Bacteria | Firmicutes | Bacilli | Lactobacillales | Streptococcaceae | Streptococcus | 796 | -3.414 | 0.811 | 2.408 | 0 | 0.001 | 0.116 | NU |
| pLD | TP2 | m17 | Bacteria | Firmicutes | Bacilli | Lactobacillales | Lactobacillaceae | Lactobacillus | 796 | 1.97 | 0.508 | 2.518 | 0 | 0.004 | 0.472 | NU |
| pLD | TP2 | m252 | Bacteria | Firmicutes | Clostridia | Clostridiales | Lachnospiraceae | Blautia | 796 | 2.048 | 0.476 | 2.652 | 0 | 0.001 | 0.076 | NU |
| pLD | TP2 | m28987 | Bacteria | Firmicutes | Bacilli | Lactobacillales | Lactobacillaceae | Lactobacillus | 796 | 1.932 | 0.497 | 2.611 | 0 | 0.004 | 0.457 | NU |
| pLD | TP2 | m3368 | Bacteria | Deferribacteres | Deferribacteres | Deferribacterales | Deferribacteraceae | Mucispirillum | 796 | -3.021 | 0.826 | 1.803 | 0 | 0.008 | 1 | NU |
| pLD | TP2 | m52033 | Bacteria | Firmicutes | Bacilli | Lactobacillales | Lactobacillaceae | Lactobacillus | 796 | 2.169 | 0.507 | 3.131 | 0 | 0.001 | 0.086 | NU |
| pLD | TP2 | m530 | Bacteria | Firmicutes | Clostridia | Clostridiales | Clostridiales_Incertae Sedis XI | Parvimonas | 796 | -7.001 | 1.945 | 1.745 | 0 | 0.009 | 1 | NU |
| pLD | TP2 | m5720 | Bacteria | Firmicutes | Clostridia | Clostridiales | Ruminococcaceae | Ruminococcus | 796 | -1.823 | 0.444 | 2.503 | 0 | 0.002 | 0.183 | NU |
| pLD | TP2 | m688 | Bacteria | Firmicutes | Clostridia | Clostridiales | Ruminococcaceae | Ruminococcus | 796 | -2.688 | 0.667 | 2.217 | 0 | 0.002 | 0.251 | NU |
| pLD | TP2 | m868 | Bacteria | Firmicutes | Clostridia | Clostridiales | Lachnospiraceae | Clostridium XlVa | 796 | -5.54 | 1.279 | 2.479 | 0 | 0.001 | 0.066 | NU |
| pLD | TP2 | m89 | Bacteria | Firmicutes | Clostridia | Clostridiales | Peptostreptococcaceae | Clostridium XI | 796 | 2.102 | 0.45 | 3.474 | 0 | 0 | 0.013 | NU |
| pADG | TP3 | m1011 | Bacteria | Firmicutes | Negativicutes | Selenomonadales | Veillonellaceae | Selenomonas | 799 | -4.19 | 0.784 | 4.213 | 0 | 0 | 0 | NU |
| pADG | TP3 | m1090 | Bacteria | Firmicutes | Clostridia | Clostridiales | Clostridiaceae 1 | Clostridium sensu stricto | 799 | -2.967 | 0.565 | 3.901 | 0 | 0 | 0.001 | NU |
| pADG | TP3 | m1178 | Bacteria | Firmicutes | Negativicutes | Selenomonadales | Veillonellaceae | Selenomonas | 799 | -5.349 | 0.919 | 4.064 | 0 | 0 | 0 | NU |
| pADG | TP3 | m122 | Bacteria | Firmicutes | Bacilli | Lactobacillales | Streptococcaceae | Streptococcus | 799 | -1.935 | 0.515 | 1.71 | 0 | 0.009 | 0.767 | NU |
| pADG | TP3 | m125 | Bacteria | Proteobacteria | Gammaproteobacteria | Aeromonadales | Succinivibrionaceae | Succinivibrio | 799 | -1.86 | 0.447 | 3.215 | 0 | 0.003 | 0.144 | NU |
| pADG | TP3 | m14 | Bacteria | Bacteroidetes | Bacteroidia | Bacteroidales | Bacteroidaceae | Bacteroides | 799 | -2.644 | 0.623 | 2.223 | 0 | 0.003 | 0.099 | NU |
| pADG | TP3 | m147 | Bacteria | Firmicutes | Clostridia | Clostridiales | Peptococcaceae 1 | Peptococcus | 799 | 1.834 | 0.285 | 5.494 | 0 | 0 | 0 | NU |
| pADG | TP3 | m149 | Bacteria | Bacteroidetes | Bacteroidia | Bacteroidales | Rikenellaceae | Alistipes | 799 | -9.876 | 2.336 | 3.173 | 0 | 0.003 | 0.106 | NU |
| pADG | TP3 | m1540 | Bacteria | Firmicutes | Clostridia | Clostridiales | Clostridiaceae 1 | Clostridium sensu stricto | 799 | -3.002 | 0.778 | 2.096 | 0 | 0.007 | 0.518 | NU |
| pADG | TP3 | m17 | Bacteria | Firmicutes | Bacilli | Lactobacillales | Lactobacillaceae | Lactobacillus | 799 | 1.889 | 0.4 | 3.182 | 0 | 0.001 | 0.01 | NU |
| pADG | TP3 | m1746 | Bacteria | Firmicutes | Clostridia | Clostridiales | Clostridiales_Incertae Sedis XI | Anaerococcus | 799 | -7.966 | 1.965 | 2.603 | 0 | 0.004 | 0.227 | NU |
| pADG | TP3 | m224 | Bacteria | Spirochaetes | Spirochaetia | Spirochaetales | Spirochaetaceae | Treponema | 799 | -2.907 | 0.657 | 2.415 | 0 | 0.002 | 0.044 | NU |
| pADG | TP3 | m255 | Bacteria | Firmicutes | Clostridia | Clostridiales | Lachnospiraceae | Roseburia | 799 | 1.637 | 0.409 | 7.003 | 0 | 0.005 | 0.277 | NU |
| pADG | TP3 | m272 | Bacteria | Firmicutes | Clostridia | Clostridiales | Clostridiales_Incertae Sedis XI | Peptoniphilus | 799 | -1.401 | 0.333 | 3.014 | 0 | 0.003 | 0.119 | NU |
| pADG | TP3 | m2739 | Bacteria | Firmicutes | Bacilli | Lactobacillales | Lactobacillaceae | Lactobacillus | 799 | 1.598 | 0.394 | 2.224 | 0 | 0.004 | 0.22 | NU |
| pADG | TP3 | m28987 | Bacteria | Firmicutes | Bacilli | Lactobacillales | Lactobacillaceae | Lactobacillus | 799 | 1.646 | 0.363 | 2.866 | 0 | 0.001 | 0.026 | NU |
| pADG | TP3 | m300 | Bacteria | Spirochaetes | Spirochaetia | Spirochaetales | Spirochaetaceae | Treponema | 799 | -1.901 | 0.448 | 2.654 | 0 | 0.003 | 0.098 | NU |
| pADG | TP3 | m44 | Bacteria | Proteobacteria | Epsilonproteobacteria | Campylobacterales | Campylobacteraceae | Campylobacter | 799 | -1.324 | 0.324 | 5.042 | 0 | 0.004 | 0.193 | NU |
| pADG | TP3 | m48895 | Bacteria | Firmicutes | Bacilli | Lactobacillales | Lactobacillaceae | Lactobacillus | 799 | 1.206 | 0.298 | 2.326 | 0 | 0.005 | 0.235 | NU |
| pADG | TP3 | m52033 | Bacteria | Firmicutes | Bacilli | Lactobacillales | Lactobacillaceae | Lactobacillus | 799 | 1.86 | 0.378 | 3.421 | 0 | 0 | 0.004 | NU |
| pADG | TP3 | m5273 | Bacteria | Firmicutes | Clostridia | Clostridiales | Lachnospiraceae | Blautia | 799 | 1.498 | 0.366 | 2.341 | 0 | 0.004 | 0.196 | NU |
| pADG | TP3 | m53 | Bacteria | Proteobacteria | Gammaproteobacteria | Aeromonadales | Succinivibrionaceae | Succinivibrio | 799 | -2.514 | 0.458 | 15.383 | 0 | 0 | 0 | NU |
| pADG | TP3 | m561 | Bacteria | Proteobacteria | Epsilonproteobacteria | Campylobacterales | Helicobacteraceae | Helicobacter | 799 | -3.308 | 0.826 | 2.007 | 0 | 0.005 | 0.28 | NU |
| pADG | TP3 | m564 | Bacteria | Firmicutes | Clostridia | Clostridiales | Lachnospiraceae | Blautia | 799 | 1.417 | 0.376 | 2.372 | 0 | 0.009 | 0.739 | NU |
| pADG | TP3 | m628 | Bacteria | Firmicutes | Clostridia | Clostridiales | Lachnospiraceae | Roseburia | 799 | 1.95 | 0.4 | 5.224 | 0 | 0 | 0.005 | NU |
| pADG | TP3 | m647 | Bacteria | Firmicutes | Erysipelotrichia | Erysipelotrichales | Erysipelotrichaceae | Erysipelotrichaceae_incertae_sedis | 799 | -7.152 | 1.663 | 2.836 | 0 | 0.003 | 0.076 | NU |
| pADG | TP3 | m665 | Bacteria | Bacteroidetes | Bacteroidia | Bacteroidales | Rikenellaceae | Alistipes | 799 | -5.917 | 1.426 | 2.392 | 0 | 0.004 | 0.151 | NU |
| pADG | TP3 | m688 | Bacteria | Firmicutes | Clostridia | Clostridiales | Ruminococcaceae | Ruminococcus | 799 | -2.847 | 0.557 | 3.316 | 0 | 0 | 0.001 | NU |
| pADG | TP3 | m69 | Bacteria | Firmicutes | Clostridia | Clostridiales | Lachnospiraceae | Dorea | 799 | -3.655 | 0.857 | 2.644 | 0 | 0.003 | 0.089 | NU |
| pADG | TP3 | m836 | Bacteria | Bacteroidetes | Bacteroidia | Bacteroidales | Bacteroidaceae | Bacteroides | 799 | -5.011 | 1.115 | 2.659 | 0 | 0.001 | 0.031 | NU |
| pADG | TP3 | m868 | Bacteria | Firmicutes | Clostridia | Clostridiales | Lachnospiraceae | Clostridium XlVa | 799 | -2.824 | 0.741 | 1.785 | 0 | 0.008 | 0.63 | NU |
| pADG | TP3 | m98 | Bacteria | Proteobacteria | Deltaproteobacteria | Desulfovibrionales | Desulfovibrionaceae | Desulfovibrio | 799 | -1.956 | 0.481 | 2.443 | 0 | 0.004 | 0.215 | NU |
| pADG | TP3 | m994 | Bacteria | Synergistetes | Synergistia | Synergistales | Synergistaceae | Cloacibacillus | 799 | -9.059 | 1.908 | 3.612 | 0 | 0.001 | 0.009 | NU |
| pBF | TP3 | m1011 | Bacteria | Firmicutes | Negativicutes | Selenomonadales | Veillonellaceae | Selenomonas | 809 | -3.436 | 0.756 | 2.8 | 0 | 0 | 0.025 | NU |
| pBF | TP3 | m10347 | Bacteria | Lentisphaerae | Oligosphaeria | Oligosphaerales | Oligosphaeraceae | Oligosphaera | 809 | -2.363 | 0.56 | 2.216 | 0 | 0.001 | 0.11 | NU |
| pBF | TP3 | m106 | Bacteria | Bacteroidetes | Bacteroidia | Bacteroidales | Bacteroidaceae | Bacteroides | 809 | -10.59 | 2.96 | 2.641 | 0 | 0.007 | 1 | NU |
| pBF | TP3 | m1069 | Bacteria | Firmicutes | Clostridia | Clostridiales | Ruminococcaceae | Ruminococcus | 809 | -3.561 | 0.815 | 2.24 | 0 | 0.001 | 0.055 | NU |
| pBF | TP3 | m1090 | Bacteria | Firmicutes | Clostridia | Clostridiales | Clostridiaceae 1 | Clostridium sensu stricto | 809 | -2.738 | 0.545 | 3.292 | 0 | 0 | 0.002 | NU |
| pBF | TP3 | m1126 | Archaea | Euryarchaeota | Methanobacteria | Methanobacteriales | Methanobacteriaceae | Methanobrevibacter | 809 | -2.745 | 0.566 | 3.025 | 0 | 0 | 0.006 | NU |
| pBF | TP3 | m1131 | Bacteria | Bacteroidetes | Bacteroidia | Bacteroidales | Porphyromonadaceae | Butyricimonas | 809 | -12.203 | 3.315 | 3.193 | 0 | 0.006 | 1 | NU |
| pBF | TP3 | m1153 | Bacteria | Synergistetes | Synergistia | Synergistales | Synergistaceae | Cloacibacillus | 809 | -10.885 | 2.977 | 2.766 | 0 | 0.006 | 1 | NU |
| pBF | TP3 | m1178 | Bacteria | Firmicutes | Negativicutes | Selenomonadales | Veillonellaceae | Selenomonas | 809 | -3.664 | 0.899 | 1.887 | 0 | 0.002 | 0.205 | NU |
| pBF | TP3 | m1180 | Bacteria | Firmicutes | Bacilli | Lactobacillales | Lactobacillaceae | Lactobacillus | 809 | 1.569 | 0.397 | 2.102 | 0 | 0.002 | 0.343 | NU |
| pBF | TP3 | m122 | Bacteria | Firmicutes | Bacilli | Lactobacillales | Streptococcaceae | Streptococcus | 809 | -2.169 | 0.459 | 2.474 | 0 | 0 | 0.011 | NU |
| pBF | TP3 | m125 | Bacteria | Proteobacteria | Gammaproteobacteria | Aeromonadales | Succinivibrionaceae | Succinivibrio | 809 | -1.786 | 0.429 | 2.946 | 0 | 0.001 | 0.14 | NU |
| pBF | TP3 | m1344 | Bacteria | Firmicutes | Clostridia | Clostridiales | Lachnospiraceae | Blautia | 809 | -0.961 | 0.266 | 1.611 | 0 | 0.007 | 1 | NU |
| pBF | TP3 | m1356 | Bacteria | Synergistetes | Synergistia | Synergistales | Synergistaceae | Pyramidobacter | 809 | -7.097 | 1.966 | 1.969 | 0 | 0.007 | 1 | NU |
| pBF | TP3 | m13690 | Bacteria | Proteobacteria | Gammaproteobacteria | Enterobacteriales | Enterobacteriaceae | Escherichia/Shigella | 809 | -1.399 | 0.332 | 1.978 | 0 | 0.001 | 0.115 | NU |
| pBF | TP3 | m14 | Bacteria | Bacteroidetes | Bacteroidia | Bacteroidales | Bacteroidaceae | Bacteroides | 809 | -2.804 | 0.574 | 2.706 | 0 | 0 | 0.005 | NU |
| pBF | TP3 | m1468 | Bacteria | Firmicutes | Bacilli | Lactobacillales | Lactobacillaceae | Lactobacillus | 809 | 2.433 | 0.571 | 2.395 | 0 | 0.001 | 0.091 | NU |
| pBF | TP3 | m147 | Bacteria | Firmicutes | Clostridia | Clostridiales | Peptococcaceae 1 | Peptococcus | 809 | 1.84 | 0.274 | 5.505 | 0 | 0 | 0 | NU |
| pBF | TP3 | m1503 | Bacteria | Firmicutes | Clostridia | Clostridiales | Clostridiaceae 1 | Clostridium sensu stricto | 809 | 1.489 | 0.413 | 2.072 | 0 | 0.007 | 1 | NU |
| pBF | TP3 | m1537 | Bacteria | Bacteroidetes | Bacteroidia | Bacteroidales | Porphyromonadaceae | Butyricimonas | 809 | -7.8 | 2.217 | 2.085 | 0 | 0.008 | 1 | NU |
| pBF | TP3 | m1540 | Bacteria | Firmicutes | Clostridia | Clostridiales | Clostridiaceae 1 | Clostridium sensu stricto | 809 | -2.958 | 0.744 | 2.058 | 0 | 0.002 | 0.313 | NU |
| pBF | TP3 | m1584 | Bacteria | Verrucomicrobia | Subdivision5 | Subdivision5_genera_incertae_sedis | Subdivision5_genera_incertae_sedis | Subdivision5_genera_incertae_sedis | 809 | -2.441 | 0.696 | 1.526 | 0 | 0.009 | 1 | NU |
| pBF | TP3 | m16 | Bacteria | Firmicutes | Clostridia | Clostridiales | Lachnospiraceae | Blautia | 809 | 1.069 | 0.274 | 2.119 | 0 | 0.003 | 0.439 | NU |
| pBF | TP3 | m17 | Bacteria | Firmicutes | Bacilli | Lactobacillales | Lactobacillaceae | Lactobacillus | 809 | 2.13 | 0.381 | 4.05 | 0 | 0 | 0 | NU |
| pBF | TP3 | m1737 | Bacteria | Firmicutes | Clostridia | Clostridiales | Ruminococcaceae | Oscillibacter | 809 | 1.528 | 0.369 | 2.192 | 0 | 0.001 | 0.157 | NU |
| pBF | TP3 | m1746 | Bacteria | Firmicutes | Clostridia | Clostridiales | Clostridiales_Incertae Sedis XI | Anaerococcus | 809 | -6.813 | 1.624 | 2.471 | 0 | 0.001 | 0.123 | NU |
| pBF | TP3 | m184 | Bacteria | Bacteroidetes | Bacteroidia | Bacteroidales | Prevotellaceae | Prevotella | 809 | 1.235 | 0.345 | 2.809 | 0 | 0.007 | 1 | NU |
| pBF | TP3 | m19 | Bacteria | Firmicutes | Clostridia | Clostridiales | Lachnospiraceae | Roseburia | 809 | 1.133 | 0.311 | 4.06 | 0 | 0.006 | 1 | NU |
| pBF | TP3 | m2023 | Bacteria | Firmicutes | Clostridia | Clostridiales | Ruminococcaceae | Butyricicoccus | 809 | 1.877 | 0.406 | 6.651 | 0 | 0 | 0.017 | NU |
| pBF | TP3 | m20914 | Bacteria | Firmicutes | Bacilli | Lactobacillales | Lactobacillaceae | Lactobacillus | 809 | 1.73 | 0.418 | 2.264 | 0 | 0.001 | 0.158 | NU |
| pBF | TP3 | m21594 | Bacteria | Synergistetes | Synergistia | Synergistales | Synergistaceae | Cloacibacillus | 809 | -9.534 | 1.915 | 3.696 | 0 | 0 | 0.003 | NU |
| pBF | TP3 | m224 | Bacteria | Spirochaetes | Spirochaetia | Spirochaetales | Spirochaetaceae | Treponema | 809 | -3.836 | 0.591 | 4.715 | 0 | 0 | 0 | NU |
| pBF | TP3 | m22914 | Bacteria | Synergistetes | Synergistia | Synergistales | Synergistaceae | Cloacibacillus | 809 | -8.083 | 2.21 | 2.231 | 0 | 0.006 | 1 | NU |
| pBF | TP3 | m23 | Bacteria | Firmicutes | Clostridia | Clostridiales | Ruminococcaceae | Faecalibacterium | 809 | 1.237 | 0.29 | 3.83 | 0 | 0.001 | 0.092 | NU |
| pBF | TP3 | m23515 | Bacteria | Bacteroidetes | Bacteroidia | Bacteroidales | Porphyromonadaceae | Butyricimonas | 809 | -9.655 | 2.42 | 2.821 | 0 | 0.002 | 0.297 | NU |
| pBF | TP3 | m2495 | Bacteria | Proteobacteria | Epsilonproteobacteria | Campylobacterales | Helicobacteraceae | Helicobacter | 809 | -2.405 | 0.679 | 1.483 | 0 | 0.008 | 1 | NU |
| pBF | TP3 | m25 | Bacteria | Proteobacteria | Epsilonproteobacteria | Campylobacterales | Campylobacteraceae | Campylobacter | 809 | -2.519 | 0.697 | 1.487 | 0 | 0.007 | 1 | NU |
| pBF | TP3 | m2542 | Bacteria | Firmicutes | Bacilli | Lactobacillales | Lactobacillaceae | Lactobacillus | 809 | 1.284 | 0.362 | 1.47 | 0 | 0.008 | 1 | NU |
| pBF | TP3 | m255 | Bacteria | Firmicutes | Clostridia | Clostridiales | Lachnospiraceae | Roseburia | 809 | 1.833 | 0.389 | 8.778 | 0 | 0 | 0.011 | NU |
| pBF | TP3 | m2570 | Bacteria | Synergistetes | Synergistia | Synergistales | Synergistaceae | Cloacibacillus | 809 | -7.619 | 2.109 | 2.057 | 0 | 0.007 | 1 | NU |
| pBF | TP3 | m258 | Bacteria | Actinobacteria | Actinobacteria | Actinomycetales | Actinomycetaceae | Actinomyces | 809 | -4.294 | 1.15 | 1.851 | 0 | 0.005 | 0.846 | NU |
| pBF | TP3 | m2631 | Bacteria | Verrucomicrobia | Subdivision5 | Subdivision5_genera_incertae_sedis | Subdivision5_genera_incertae_sedis | Subdivision5_genera_incertae_sedis | 809 | -3.686 | 0.915 | 1.871 | 0 | 0.002 | 0.255 | NU |
| pBF | TP3 | m272 | Bacteria | Firmicutes | Clostridia | Clostridiales | Clostridiales_Incertae Sedis XI | Peptoniphilus | 809 | -1.199 | 0.317 | 2.257 | 0 | 0.004 | 0.684 | NU |
| pBF | TP3 | m2739 | Bacteria | Firmicutes | Bacilli | Lactobacillales | Lactobacillaceae | Lactobacillus | 809 | 1.536 | 0.378 | 2.062 | 0 | 0.002 | 0.215 | NU |
| pBF | TP3 | m277 | Bacteria | Firmicutes | Erysipelotrichia | Erysipelotrichales | Erysipelotrichaceae | Bulleidia | 809 | 2.025 | 0.448 | 2.599 | 0 | 0 | 0.028 | NU |
| pBF | TP3 | m28987 | Bacteria | Firmicutes | Bacilli | Lactobacillales | Lactobacillaceae | Lactobacillus | 809 | 1.933 | 0.346 | 3.953 | 0 | 0 | 0 | NU |
| pBF | TP3 | m294 | Bacteria | Firmicutes | Clostridia | Clostridiales | Lachnospiraceae | Roseburia | 809 | 1.859 | 0.354 | 5.378 | 0 | 0 | 0.001 | NU |
| pBF | TP3 | m295 | Archaea | Euryarchaeota | Methanobacteria | Methanobacteriales | Methanobacteriaceae | Methanobrevibacter | 809 | -2.242 | 0.465 | 3.002 | 0 | 0 | 0.006 | NU |
| pBF | TP3 | m300 | Bacteria | Spirochaetes | Spirochaetia | Spirochaetales | Spirochaetaceae | Treponema | 809 | -2.159 | 0.428 | 3.414 | 0 | 0 | 0.002 | NU |
| pBF | TP3 | m301 | Bacteria | Firmicutes | Clostridia | Clostridiales | Peptostreptococcaceae | Peptostreptococcus | 809 | -2.085 | 0.501 | 1.995 | 0 | 0.001 | 0.142 | NU |
| pBF | TP3 | m307 | Bacteria | Bacteroidetes | Bacteroidia | Bacteroidales | Porphyromonadaceae | Parabacteroides | 809 | -5.24 | 1.492 | 1.735 | 0 | 0.008 | 1 | NU |
| pBF | TP3 | m325 | Bacteria | Firmicutes | Clostridia | Clostridiales | Lachnospiraceae | Roseburia | 809 | 1.792 | 0.395 | 6.258 | 0 | 0 | 0.025 | NU |
| pBF | TP3 | m326 | Bacteria | Firmicutes | Clostridia | Clostridiales | Lachnospiraceae | Pseudobutyrivibrio | 809 | 1.964 | 0.368 | 5.067 | 0 | 0 | 0 | NU |
| pBF | TP3 | m327 | Archaea | Euryarchaeota | Methanobacteria | Methanobacteriales | Methanobacteriaceae | Methanobrevibacter | 809 | -1.226 | 0.33 | 2.232 | 0 | 0.005 | 0.908 | NU |
| pBF | TP3 | m3271 | Bacteria | Firmicutes | Bacilli | Lactobacillales | Lactobacillaceae | Lactobacillus | 809 | 1.111 | 0.319 | 1.424 | 0 | 0.009 | 1 | NU |
| pBF | TP3 | m35073 | Bacteria | Firmicutes | Bacilli | Lactobacillales | Lactobacillaceae | Lactobacillus | 809 | 1.6 | 0.288 | 4.012 | 0 | 0 | 0 | NU |
| pBF | TP3 | m3686 | Bacteria | Bacteroidetes | Bacteroidia | Bacteroidales | Rikenellaceae | Alistipes | 809 | -5.616 | 1.457 | 1.774 | 0 | 0.003 | 0.523 | NU |
| pBF | TP3 | m37847 | Bacteria | Firmicutes | Clostridia | Clostridiales | Ruminococcaceae | Faecalibacterium | 809 | 1.121 | 0.321 | 2.759 | 0 | 0.009 | 1 | NU |
| pBF | TP3 | m3949 | Bacteria | Fusobacteria | Fusobacteriia | Fusobacteriales | Fusobacteriaceae | Fusobacterium | 809 | -8.283 | 2.027 | 2.817 | 0 | 0.002 | 0.198 | NU |
| pBF | TP3 | m40 | Bacteria | Firmicutes | Clostridia | Clostridiales | Clostridiaceae 1 | Clostridium sensu stricto | 809 | -2.175 | 0.615 | 3.745 | 0 | 0.008 | 1 | NU |
| pBF | TP3 | m411 | Bacteria | Firmicutes | Bacilli | Lactobacillales | Lactobacillaceae | Lactobacillus | 809 | 0.995 | 0.275 | 1.959 | 0 | 0.007 | 1 | NU |
| pBF | TP3 | m425 | Bacteria | Firmicutes | Clostridia | Clostridiales | Lachnospiraceae | Blautia | 809 | 2.079 | 0.39 | 3.836 | 0 | 0 | 0 | NU |
| pBF | TP3 | m432 | Bacteria | Firmicutes | Clostridia | Clostridiales | Lachnospiraceae | Blautia | 809 | 1.267 | 0.292 | 2.589 | 0 | 0.001 | 0.064 | NU |
| pBF | TP3 | m45649 | Bacteria | Proteobacteria | Gammaproteobacteria | Enterobacteriales | Enterobacteriaceae | Escherichia/Shigella | 809 | -2.165 | 0.488 | 2.221 | 0 | 0.001 | 0.041 | NU |
| pBF | TP3 | m472 | Bacteria | Firmicutes | Clostridia | Clostridiales | Lachnospiraceae | Blautia | 809 | 1.661 | 0.367 | 3.266 | 0 | 0 | 0.027 | NU |
| pBF | TP3 | m48895 | Bacteria | Firmicutes | Bacilli | Lactobacillales | Lactobacillaceae | Lactobacillus | 809 | 1.71 | 0.283 | 4.65 | 0 | 0 | 0 | NU |
| pBF | TP3 | m50608 | Bacteria | Firmicutes | Bacilli | Lactobacillales | Lactobacillaceae | Lactobacillus | 809 | 2.062 | 0.511 | 2.02 | 0 | 0.002 | 0.243 | NU |
| pBF | TP3 | m52033 | Bacteria | Firmicutes | Bacilli | Lactobacillales | Lactobacillaceae | Lactobacillus | 809 | 2.055 | 0.361 | 4.178 | 0 | 0 | 0 | NU |
| pBF | TP3 | m5273 | Bacteria | Firmicutes | Clostridia | Clostridiales | Lachnospiraceae | Blautia | 809 | 1.644 | 0.352 | 2.811 | 0 | 0 | 0.013 | NU |
| pBF | TP3 | m53 | Bacteria | Proteobacteria | Gammaproteobacteria | Aeromonadales | Succinivibrionaceae | Succinivibrio | 809 | -2.642 | 0.439 | 16.999 | 0 | 0 | 0 | NU |
| pBF | TP3 | m530 | Bacteria | Firmicutes | Clostridia | Clostridiales | Clostridiales_Incertae Sedis XI | Parvimonas | 809 | -3.059 | 0.863 | 1.526 | 0 | 0.008 | 1 | NU |
| pBF | TP3 | m561 | Bacteria | Proteobacteria | Epsilonproteobacteria | Campylobacterales | Helicobacteraceae | Helicobacter | 809 | -3.036 | 0.795 | 1.683 | 0 | 0.004 | 0.606 | NU |
| pBF | TP3 | m564 | Bacteria | Firmicutes | Clostridia | Clostridiales | Lachnospiraceae | Blautia | 809 | 1.867 | 0.358 | 4.103 | 0 | 0 | 0.001 | NU |
| pBF | TP3 | m567 | Bacteria | Fusobacteria | Fusobacteriia | Fusobacteriales | Fusobacteriaceae | Fusobacterium | 809 | -4.202 | 1.2 | 1.623 | 0 | 0.009 | 1 | NU |
| pBF | TP3 | m5720 | Bacteria | Firmicutes | Clostridia | Clostridiales | Ruminococcaceae | Ruminococcus | 809 | -4.849 | 1.338 | 1.658 | 0 | 0.007 | 1 | NU |
| pBF | TP3 | m58 | Bacteria | Firmicutes | Clostridia | Clostridiales | Ruminococcaceae | Ruminococcus | 809 | 1.041 | 0.279 | 2.237 | 0 | 0.005 | 0.875 | NU |
| pBF | TP3 | m595 | Bacteria | Firmicutes | Clostridia | Clostridiales | Lachnospiraceae | Blautia | 809 | 2.08 | 0.32 | 5.407 | 0 | 0 | 0 | NU |
| pBF | TP3 | m647 | Bacteria | Firmicutes | Erysipelotrichia | Erysipelotrichales | Erysipelotrichaceae | Erysipelotrichaceae_incertae_sedis | 809 | -6.442 | 1.599 | 2.299 | 0 | 0.002 | 0.252 | NU |
| pBF | TP3 | m68 | Bacteria | Bacteroidetes | Bacteroidia | Bacteroidales | Prevotellaceae | Prevotella | 809 | -1.946 | 0.492 | 2.574 | 0 | 0.002 | 0.347 | NU |
| pBF | TP3 | m688 | Bacteria | Firmicutes | Clostridia | Clostridiales | Ruminococcaceae | Ruminococcus | 809 | -2.876 | 0.519 | 3.558 | 0 | 0 | 0 | NU |
| pBF | TP3 | m69 | Bacteria | Firmicutes | Clostridia | Clostridiales | Lachnospiraceae | Dorea | 809 | -3.235 | 0.827 | 2.053 | 0 | 0.003 | 0.414 | NU |
| pBF | TP3 | m761 | Bacteria | Synergistetes | Synergistia | Synergistales | Synergistaceae | Cloacibacillus | 809 | -7.826 | 2.144 | 2.167 | 0 | 0.006 | 1 | NU |
| pBF | TP3 | m80 | Bacteria | Firmicutes | Clostridia | Clostridiales | Ruminococcaceae | Butyricicoccus | 809 | -1.277 | 0.332 | 2.651 | 0 | 0.004 | 0.548 | NU |
| pBF | TP3 | m801 | Bacteria | Proteobacteria | Gammaproteobacteria | Aeromonadales | Succinivibrionaceae | Succinivibrio | 809 | -2.035 | 0.582 | 1.883 | 0 | 0.009 | 1 | NU |
| pBF | TP3 | m836 | Bacteria | Bacteroidetes | Bacteroidia | Bacteroidales | Bacteroidaceae | Bacteroides | 809 | -4.361 | 1.038 | 2.131 | 0 | 0.001 | 0.118 | NU |
| pBF | TP3 | m851 | Bacteria | Lentisphaerae | Oligosphaeria | Oligosphaerales | Oligosphaeraceae | Oligosphaera | 809 | -3.752 | 1.084 | 1.384 | 0 | 0.009 | 1 | NU |
| pBF | TP3 | m87 | Bacteria | Firmicutes | Bacilli | Lactobacillales | Lactobacillaceae | Lactobacillus | 809 | 0.978 | 0.283 | 1.505 | 0 | 0.01 | 1 | NU |
| pBF | TP3 | m88 | Bacteria | Firmicutes | Clostridia | Clostridiales | Lachnospiraceae | Coprococcus | 809 | 1.712 | 0.387 | 6.359 | 0 | 0.001 | 0.044 | NU |
| pBF | TP3 | m8837 | Bacteria | Firmicutes | Clostridia | Clostridiales | Lachnospiraceae | Blautia | 809 | 1.171 | 0.337 | 1.544 | 0 | 0.009 | 1 | NU |
| pBF | TP3 | m910 | Bacteria | Firmicutes | Bacilli | Lactobacillales | Streptococcaceae | Streptococcus | 809 | 1.374 | 0.363 | 2.188 | 0 | 0.004 | 0.695 | NU |
| pBF | TP3 | m955 | Bacteria | Firmicutes | Clostridia | Clostridiales | Lachnospiraceae | Roseburia | 809 | 1.757 | 0.501 | 1.61 | 0 | 0.009 | 1 | NU |
| pBF | TP3 | m98 | Bacteria | Proteobacteria | Deltaproteobacteria | Desulfovibrionales | Desulfovibrionaceae | Desulfovibrio | 809 | -1.843 | 0.457 | 2.207 | 0 | 0.002 | 0.252 | NU |
| pBF | TP3 | m994 | Bacteria | Synergistetes | Synergistia | Synergistales | Synergistaceae | Cloacibacillus | 809 | -7.934 | 1.814 | 2.793 | 0 | 0.001 | 0.055 | NU |
| pLA | TP3 | m1011 | Bacteria | Firmicutes | Negativicutes | Selenomonadales | Veillonellaceae | Selenomonas | 809 | -4.088 | 0.846 | 3.964 | 0 | 0 | 0.006 | NU |
| pLA | TP3 | m106 | Bacteria | Bacteroidetes | Bacteroidia | Bacteroidales | Bacteroidaceae | Bacteroides | 809 | -16.481 | 3.286 | 6.396 | 0 | 0 | 0.002 | NU |
| pLA | TP3 | m1090 | Bacteria | Firmicutes | Clostridia | Clostridiales | Clostridiaceae 1 | Clostridium sensu stricto | 809 | -2.734 | 0.612 | 3.28 | 0 | 0.001 | 0.036 | NU |
| pLA | TP3 | m1109 | Bacteria | Firmicutes | Bacilli | Lactobacillales | Streptococcaceae | Streptococcus | 809 | -5.242 | 1.158 | 3.105 | 0 | 0.001 | 0.027 | NU |
| pLA | TP3 | m1126 | Archaea | Euryarchaeota | Methanobacteria | Methanobacteriales | Methanobacteriaceae | Methanobrevibacter | 809 | -2.788 | 0.634 | 3.119 | 0 | 0.001 | 0.05 | NU |
| pLA | TP3 | m1131 | Bacteria | Bacteroidetes | Bacteroidia | Bacteroidales | Porphyromonadaceae | Butyricimonas | 809 | -17.157 | 3.692 | 6.312 | 0 | 0 | 0.015 | NU |
| pLA | TP3 | m114 | Bacteria | Bacteroidetes | Bacteroidia | Bacteroidales | Porphyromonadaceae | Parabacteroides | 809 | -9.001 | 1.605 | 5.206 | 0 | 0 | 0 | NU |
| pLA | TP3 | m1178 | Bacteria | Firmicutes | Negativicutes | Selenomonadales | Veillonellaceae | Selenomonas | 809 | -3.974 | 1.009 | 2.219 | 0 | 0.004 | 0.371 | NU |
| pLA | TP3 | m122 | Bacteria | Firmicutes | Bacilli | Lactobacillales | Streptococcaceae | Streptococcus | 809 | -1.871 | 0.518 | 1.841 | 0 | 0.01 | 1 | NU |
| pLA | TP3 | m1282 | Bacteria | Firmicutes | Clostridia | Clostridiales | Ruminococcaceae | Butyricicoccus | 809 | -7.969 | 1.703 | 3.694 | 0 | 0 | 0.013 | NU |
| pLA | TP3 | m1356 | Bacteria | Synergistetes | Synergistia | Synergistales | Synergistaceae | Pyramidobacter | 809 | -11.283 | 2.182 | 4.977 | 0 | 0 | 0.001 | NU |
| pLA | TP3 | m14 | Bacteria | Bacteroidetes | Bacteroidia | Bacteroidales | Bacteroidaceae | Bacteroides | 809 | -2.963 | 0.644 | 3.022 | 0 | 0 | 0.019 | NU |
| pLA | TP3 | m147 | Bacteria | Firmicutes | Clostridia | Clostridiales | Peptococcaceae 1 | Peptococcus | 809 | 1.461 | 0.311 | 3.47 | 0 | 0 | 0.012 | NU |
| pLA | TP3 | m149 | Bacteria | Bacteroidetes | Bacteroidia | Bacteroidales | Rikenellaceae | Alistipes | 809 | -9.909 | 2.533 | 3.179 | 0 | 0.004 | 0.413 | NU |
| pLA | TP3 | m1537 | Bacteria | Bacteroidetes | Bacteroidia | Bacteroidales | Porphyromonadaceae | Butyricimonas | 809 | -13.716 | 2.453 | 6.447 | 0 | 0 | 0 | NU |
| pLA | TP3 | m1746 | Bacteria | Firmicutes | Clostridia | Clostridiales | Clostridiales_Incertae Sedis XI | Anaerococcus | 809 | -11.277 | 1.794 | 6.769 | 0 | 0 | 0 | NU |
| pLA | TP3 | m179 | Bacteria | Bacteroidetes | Bacteroidia | Bacteroidales | Porphyromonadaceae | Butyricimonas | 809 | -15.339 | 2.932 | 6.582 | 0 | 0 | 0.001 | NU |
| pLA | TP3 | m21594 | Bacteria | Synergistetes | Synergistia | Synergistales | Synergistaceae | Cloacibacillus | 809 | -9.834 | 2.151 | 3.932 | 0 | 0 | 0.022 | NU |
| pLA | TP3 | m224 | Bacteria | Spirochaetes | Spirochaetia | Spirochaetales | Spirochaetaceae | Treponema | 809 | -2.994 | 0.669 | 2.873 | 0 | 0.001 | 0.034 | NU |
| pLA | TP3 | m22914 | Bacteria | Synergistetes | Synergistia | Synergistales | Synergistaceae | Cloacibacillus | 809 | -12.021 | 2.453 | 4.936 | 0 | 0 | 0.004 | NU |
| pLA | TP3 | m23515 | Bacteria | Bacteroidetes | Bacteroidia | Bacteroidales | Porphyromonadaceae | Butyricimonas | 809 | -15.513 | 2.679 | 7.283 | 0 | 0 | 0 | NU |
| pLA | TP3 | m2495 | Bacteria | Proteobacteria | Epsilonproteobacteria | Campylobacterales | Helicobacteraceae | Helicobacter | 809 | -2.744 | 0.758 | 1.931 | 0 | 0.009 | 1 | NU |
| pLA | TP3 | m255 | Bacteria | Firmicutes | Clostridia | Clostridiales | Lachnospiraceae | Roseburia | 809 | 1.924 | 0.433 | 9.674 | 0 | 0.001 | 0.041 | NU |
| pLA | TP3 | m2570 | Bacteria | Synergistetes | Synergistia | Synergistales | Synergistaceae | Cloacibacillus | 809 | -12.077 | 2.34 | 5.168 | 0 | 0 | 0.001 | NU |
| pLA | TP3 | m2631 | Bacteria | Verrucomicrobia | Subdivision5 | Subdivision5_genera_incertae_sedis | Subdivision5_genera_incertae_sedis | Subdivision5_genera_incertae_sedis | 809 | -4.318 | 1.027 | 2.567 | 0 | 0.001 | 0.117 | NU |
| pLA | TP3 | m272 | Bacteria | Firmicutes | Clostridia | Clostridiales | Clostridiales_Incertae Sedis XI | Peptoniphilus | 809 | -1.419 | 0.354 | 3.159 | 0 | 0.003 | 0.274 | NU |
| pLA | TP3 | m277 | Bacteria | Firmicutes | Erysipelotrichia | Erysipelotrichales | Erysipelotrichaceae | Bulleidia | 809 | 1.835 | 0.504 | 2.136 | 0 | 0.009 | 1 | NU |
| pLA | TP3 | m2793 | Bacteria | Proteobacteria | Deltaproteobacteria | Desulfovibrionales | Desulfovibrionaceae | Desulfovibrio | 809 | -7.398 | 1.855 | 2.641 | 0 | 0.003 | 0.301 | NU |
| pLA | TP3 | m3 | Bacteria | Proteobacteria | Gammaproteobacteria | Enterobacteriales | Enterobacteriaceae | Escherichia/Shigella | 809 | -10.606 | 2.577 | 3.734 | 0 | 0.002 | 0.174 | NU |
| pLA | TP3 | m301 | Bacteria | Firmicutes | Clostridia | Clostridiales | Peptostreptococcaceae | Peptostreptococcus | 809 | -2.514 | 0.56 | 2.899 | 0 | 0.001 | 0.032 | NU |
| pLA | TP3 | m3165 | Bacteria | Firmicutes | Clostridia | Clostridiales | Peptostreptococcaceae | Clostridium XI | 809 | 1.212 | 0.331 | 2.76 | 0 | 0.008 | 1 | NU |
| pLA | TP3 | m326 | Bacteria | Firmicutes | Clostridia | Clostridiales | Lachnospiraceae | Pseudobutyrivibrio | 809 | 1.518 | 0.416 | 3.026 | 0 | 0.009 | 1 | NU |
| pLA | TP3 | m333 | Bacteria | Bacteroidetes | Bacteroidia | Bacteroidales | Porphyromonadaceae | Parabacteroides | 809 | -5.834 | 1.281 | 3.147 | 0 | 0 | 0.024 | NU |
| pLA | TP3 | m34303 | Bacteria | Bacteroidetes | Bacteroidia | Bacteroidales | Bacteroidaceae | Bacteroides | 809 | -2.949 | 0.763 | 2.2 | 0 | 0.005 | 0.499 | NU |
| pLA | TP3 | m365 | Bacteria | Bacteroidetes | Bacteroidia | Bacteroidales | Prevotellaceae | Alloprevotella | 809 | -9.776 | 2.243 | 3.444 | 0 | 0.001 | 0.059 | NU |
| pLA | TP3 | m3949 | Bacteria | Fusobacteria | Fusobacteriia | Fusobacteriales | Fusobacteriaceae | Fusobacterium | 809 | -10.199 | 2.268 | 4.272 | 0 | 0.001 | 0.031 | NU |
| pLA | TP3 | m44 | Bacteria | Proteobacteria | Epsilonproteobacteria | Campylobacterales | Campylobacteraceae | Campylobacter | 809 | -1.425 | 0.344 | 5.83 | 0 | 0.002 | 0.155 | NU |
| pLA | TP3 | m463 | Bacteria | Firmicutes | Clostridia | Clostridiales | Ruminococcaceae | Intestinimonas | 809 | -6.367 | 1.191 | 4.403 | 0 | 0 | 0 | NU |
| pLA | TP3 | m477 | Bacteria | Firmicutes | Clostridia | Clostridiales | Peptococcaceae 1 | Peptococcus | 809 | -1.465 | 0.383 | 2.582 | 0 | 0.005 | 0.594 | NU |
| pLA | TP3 | m4827 | Bacteria | Bacteroidetes | Bacteroidia | Bacteroidales | Porphyromonadaceae | Porphyromonas | 809 | -3.761 | 1.01 | 2.146 | 0 | 0.007 | 0.882 | NU |
| pLA | TP3 | m52033 | Bacteria | Firmicutes | Bacilli | Lactobacillales | Lactobacillaceae | Lactobacillus | 809 | 1.501 | 0.409 | 2.229 | 0 | 0.008 | 1 | NU |
| pLA | TP3 | m5273 | Bacteria | Firmicutes | Clostridia | Clostridiales | Lachnospiraceae | Blautia | 809 | 1.459 | 0.395 | 2.214 | 0 | 0.008 | 0.996 | NU |
| pLA | TP3 | m53 | Bacteria | Proteobacteria | Gammaproteobacteria | Aeromonadales | Succinivibrionaceae | Succinivibrio | 809 | -2.544 | 0.492 | 15.756 | 0 | 0 | 0.001 | NU |
| pLA | TP3 | m530 | Bacteria | Firmicutes | Clostridia | Clostridiales | Clostridiales_Incertae Sedis XI | Parvimonas | 809 | -4.345 | 0.963 | 3.079 | 0 | 0.001 | 0.029 | NU |
| pLA | TP3 | m535 | Bacteria | Actinobacteria | Actinobacteria | Bifidobacteriales | Bifidobacteriaceae | Bifidobacterium | 809 | -3.877 | 0.985 | 2.432 | 0 | 0.004 | 0.372 | NU |
| pLA | TP3 | m567 | Bacteria | Fusobacteria | Fusobacteriia | Fusobacteriales | Fusobacteriaceae | Fusobacterium | 809 | -5.541 | 1.341 | 2.822 | 0 | 0.002 | 0.163 | NU |
| pLA | TP3 | m628 | Bacteria | Firmicutes | Clostridia | Clostridiales | Lachnospiraceae | Roseburia | 809 | 1.925 | 0.43 | 5.056 | 0 | 0.001 | 0.034 | NU |
| pLA | TP3 | m638 | Bacteria | Bacteroidetes | Bacteroidia | Bacteroidales | Bacteroidaceae | Bacteroides | 809 | -7.4 | 1.637 | 3.291 | 0 | 0.001 | 0.028 | NU |
| pLA | TP3 | m647 | Bacteria | Firmicutes | Erysipelotrichia | Erysipelotrichales | Erysipelotrichaceae | Erysipelotrichaceae_incertae_sedis | 809 | -6.489 | 1.796 | 2.333 | 0 | 0.01 | 1 | NU |
| pLA | TP3 | m664 | Bacteria | Firmicutes | Clostridia | Clostridiales | Clostridiales_Incertae Sedis XI | Anaerococcus | 809 | -1.578 | 0.394 | 2.564 | 0 | 0.003 | 0.28 | NU |
| pLA | TP3 | m665 | Bacteria | Bacteroidetes | Bacteroidia | Bacteroidales | Rikenellaceae | Alistipes | 809 | -7.457 | 1.538 | 3.765 | 0 | 0 | 0.006 | NU |
| pLA | TP3 | m688 | Bacteria | Firmicutes | Clostridia | Clostridiales | Ruminococcaceae | Ruminococcus | 809 | -2.495 | 0.585 | 2.678 | 0 | 0.001 | 0.091 | NU |
| pLA | TP3 | m69 | Bacteria | Firmicutes | Clostridia | Clostridiales | Lachnospiraceae | Dorea | 809 | -3.435 | 0.926 | 2.313 | 0 | 0.008 | 0.937 | NU |
| pLA | TP3 | m743 | Bacteria | Firmicutes | Clostridia | Clostridiales | Lachnospiraceae | Howardella | 809 | -7.69 | 1.92 | 3.075 | 0 | 0.003 | 0.279 | NU |
| pLA | TP3 | m801 | Bacteria | Proteobacteria | Gammaproteobacteria | Aeromonadales | Succinivibrionaceae | Succinivibrio | 809 | -2.493 | 0.648 | 2.826 | 0 | 0.005 | 0.543 | NU |
| pLA | TP3 | m82 | Bacteria | Firmicutes | Bacilli | Lactobacillales | Enterococcaceae | Enterococcus | 809 | -3.881 | 0.789 | 3.501 | 0 | 0 | 0.004 | NU |
| pLA | TP3 | m836 | Bacteria | Bacteroidetes | Bacteroidia | Bacteroidales | Bacteroidaceae | Bacteroides | 809 | -6.337 | 1.153 | 4.499 | 0 | 0 | 0 | NU |
| pLA | TP3 | m854 | Bacteria | Bacteroidetes | Bacteroidia | Bacteroidales | Rikenellaceae | Alistipes | 809 | -21.288 | 5.875 | 6.976 | 0 | 0.009 | 1 | NU |
| pLA | TP3 | m971 | Bacteria | Firmicutes | Clostridia | Clostridiales | Clostridiales_Incertae Sedis XI | Helcococcus | 809 | -6.851 | 1.812 | 2.737 | 0 | 0.006 | 0.703 | NU |
| pLA | TP3 | m977 | Bacteria | Actinobacteria | Actinobacteria | Coriobacteriales | Coriobacteriaceae | Atopobium | 809 | -3.7 | 0.848 | 2.813 | 0 | 0.001 | 0.057 | NU |
| pLD | TP3 | m1011 | Bacteria | Firmicutes | Negativicutes | Selenomonadales | Veillonellaceae | Selenomonas | 809 | -3.464 | 0.817 | 2.847 | 0 | 0.001 | 0.099 | NU |
| pLD | TP3 | m10347 | Bacteria | Lentisphaerae | Oligosphaeria | Oligosphaerales | Oligosphaeraceae | Oligosphaera | 809 | -2.343 | 0.604 | 2.178 | 0 | 0.004 | 0.477 | NU |
| pLD | TP3 | m106 | Bacteria | Bacteroidetes | Bacteroidia | Bacteroidales | Bacteroidaceae | Bacteroides | 809 | -14.137 | 3.175 | 4.707 | 0 | 0.001 | 0.038 | NU |
| pLD | TP3 | m1090 | Bacteria | Firmicutes | Clostridia | Clostridiales | Clostridiaceae 1 | Clostridium sensu stricto | 809 | -2.262 | 0.591 | 2.247 | 0 | 0.005 | 0.579 | NU |
| pLD | TP3 | m1109 | Bacteria | Firmicutes | Bacilli | Lactobacillales | Streptococcaceae | Streptococcus | 809 | -5.028 | 1.114 | 2.858 | 0 | 0.001 | 0.028 | NU |
| pLD | TP3 | m1126 | Archaea | Euryarchaeota | Methanobacteria | Methanobacteriales | Methanobacteriaceae | Methanobrevibacter | 809 | -2.847 | 0.61 | 3.254 | 0 | 0 | 0.014 | NU |
| pLD | TP3 | m1131 | Bacteria | Bacteroidetes | Bacteroidia | Bacteroidales | Porphyromonadaceae | Butyricimonas | 809 | -15.958 | 3.557 | 5.461 | 0 | 0.001 | 0.033 | NU |
| pLD | TP3 | m114 | Bacteria | Bacteroidetes | Bacteroidia | Bacteroidales | Porphyromonadaceae | Parabacteroides | 809 | -8.915 | 1.539 | 5.108 | 0 | 0 | 0 | NU |
| pLD | TP3 | m1165 | Archaea | Euryarchaeota | Thermoplasmata | Methanomassiliicoccales | Methanomassiliicoccaceae | Methanomassiliicoccus | 809 | -2.878 | 0.791 | 1.761 | 0 | 0.008 | 1 | NU |
| pLD | TP3 | m1178 | Bacteria | Firmicutes | Negativicutes | Selenomonadales | Veillonellaceae | Selenomonas | 809 | -3.6 | 0.97 | 1.821 | 0 | 0.007 | 0.928 | NU |
| pLD | TP3 | m1204 | Bacteria | Firmicutes | Erysipelotrichia | Erysipelotrichales | Erysipelotrichaceae | Solobacterium | 809 | -7.867 | 1.923 | 2.87 | 0 | 0.002 | 0.194 | NU |
| pLD | TP3 | m124 | Bacteria | Firmicutes | Clostridia | Clostridiales | Ruminococcaceae | Oscillibacter | 809 | -2.833 | 0.788 | 1.825 | 0 | 0.009 | 1 | NU |
| pLD | TP3 | m1255 | Bacteria | Firmicutes | Clostridia | Clostridiales | Clostridiales_Incertae Sedis XI | Peptoniphilus | 809 | -2.071 | 0.478 | 2.762 | 0 | 0.001 | 0.067 | NU |
| pLD | TP3 | m1282 | Bacteria | Firmicutes | Clostridia | Clostridiales | Ruminococcaceae | Butyricicoccus | 809 | -8.078 | 1.631 | 3.796 | 0 | 0 | 0.003 | NU |
| pLD | TP3 | m1356 | Bacteria | Synergistetes | Synergistia | Synergistales | Synergistaceae | Pyramidobacter | 809 | -9.699 | 2.107 | 3.678 | 0 | 0 | 0.019 | NU |
| pLD | TP3 | m14 | Bacteria | Bacteroidetes | Bacteroidia | Bacteroidales | Bacteroidaceae | Bacteroides | 809 | -3 | 0.617 | 3.096 | 0 | 0 | 0.005 | NU |
| pLD | TP3 | m147 | Bacteria | Firmicutes | Clostridia | Clostridiales | Peptococcaceae 1 | Peptococcus | 809 | 1.396 | 0.3 | 3.171 | 0 | 0 | 0.014 | NU |
| pLD | TP3 | m149 | Bacteria | Bacteroidetes | Bacteroidia | Bacteroidales | Rikenellaceae | Alistipes | 809 | -8.941 | 2.436 | 2.588 | 0 | 0.007 | 1 | NU |
| pLD | TP3 | m15 | Bacteria | Bacteroidetes | Bacteroidia | Bacteroidales | Bacteroidaceae | Bacteroides | 809 | -2.633 | 0.672 | 2.014 | 0 | 0.004 | 0.4 | NU |
| pLD | TP3 | m1537 | Bacteria | Bacteroidetes | Bacteroidia | Bacteroidales | Porphyromonadaceae | Butyricimonas | 809 | -12.524 | 2.362 | 5.375 | 0 | 0 | 0.001 | NU |
| pLD | TP3 | m1746 | Bacteria | Firmicutes | Clostridia | Clostridiales | Clostridiales_Incertae Sedis XI | Anaerococcus | 809 | -11.112 | 1.721 | 6.574 | 0 | 0 | 0 | NU |
| pLD | TP3 | m179 | Bacteria | Bacteroidetes | Bacteroidia | Bacteroidales | Porphyromonadaceae | Butyricimonas | 809 | -13.907 | 2.825 | 5.41 | 0 | 0 | 0.004 | NU |
| pLD | TP3 | m211 | Bacteria | Firmicutes | Clostridia | Clostridiales | Clostridiales_Incertae Sedis XI | Anaerococcus | 809 | -1.336 | 0.353 | 2.31 | 0 | 0.005 | 0.696 | NU |
| pLD | TP3 | m21594 | Bacteria | Synergistetes | Synergistia | Synergistales | Synergistaceae | Cloacibacillus | 809 | -8.399 | 2.075 | 2.868 | 0 | 0.002 | 0.232 | NU |
| pLD | TP3 | m224 | Bacteria | Spirochaetes | Spirochaetia | Spirochaetales | Spirochaetaceae | Treponema | 809 | -3.07 | 0.644 | 3.021 | 0 | 0 | 0.009 | NU |
| pLD | TP3 | m22914 | Bacteria | Synergistetes | Synergistia | Synergistales | Synergistaceae | Cloacibacillus | 809 | -9.726 | 2.376 | 3.231 | 0 | 0.002 | 0.191 | NU |
| pLD | TP3 | m23515 | Bacteria | Bacteroidetes | Bacteroidia | Bacteroidales | Porphyromonadaceae | Butyricimonas | 809 | -14.607 | 2.578 | 6.457 | 0 | 0 | 0 | NU |
| pLD | TP3 | m2495 | Bacteria | Proteobacteria | Epsilonproteobacteria | Campylobacterales | Helicobacteraceae | Helicobacter | 809 | -2.771 | 0.731 | 1.97 | 0 | 0.005 | 0.675 | NU |
| pLD | TP3 | m255 | Bacteria | Firmicutes | Clostridia | Clostridiales | Lachnospiraceae | Roseburia | 809 | 1.899 | 0.419 | 9.424 | 0 | 0.001 | 0.026 | NU |
| pLD | TP3 | m2570 | Bacteria | Synergistetes | Synergistia | Synergistales | Synergistaceae | Cloacibacillus | 809 | -10.378 | 2.261 | 3.816 | 0 | 0 | 0.02 | NU |
| pLD | TP3 | m258 | Bacteria | Actinobacteria | Actinobacteria | Actinomycetales | Actinomycetaceae | Actinomyces | 809 | -4.481 | 1.238 | 2.016 | 0 | 0.008 | 1 | NU |
| pLD | TP3 | m2631 | Bacteria | Verrucomicrobia | Subdivision5 | Subdivision5_genera_incertae_sedis | Subdivision5_genera_incertae_sedis | Subdivision5_genera_incertae_sedis | 809 | -3.817 | 0.987 | 2.006 | 0 | 0.004 | 0.497 | NU |
| pLD | TP3 | m272 | Bacteria | Firmicutes | Clostridia | Clostridiales | Clostridiales_Incertae Sedis XI | Peptoniphilus | 809 | -1.497 | 0.34 | 3.516 | 0 | 0.001 | 0.048 | NU |
| pLD | TP3 | m277 | Bacteria | Firmicutes | Erysipelotrichia | Erysipelotrichales | Erysipelotrichaceae | Bulleidia | 809 | 1.817 | 0.485 | 2.094 | 0 | 0.006 | 0.803 | NU |
| pLD | TP3 | m2793 | Bacteria | Proteobacteria | Deltaproteobacteria | Desulfovibrionales | Desulfovibrionaceae | Desulfovibrio | 809 | -7.164 | 1.781 | 2.477 | 0 | 0.002 | 0.26 | NU |
| pLD | TP3 | m295 | Archaea | Euryarchaeota | Methanobacteria | Methanobacteriales | Methanobacteriaceae | Methanobrevibacter | 809 | -2.179 | 0.503 | 2.835 | 0 | 0.001 | 0.067 | NU |
| pLD | TP3 | m3 | Bacteria | Proteobacteria | Gammaproteobacteria | Enterobacteriales | Enterobacteriaceae | Escherichia/Shigella | 809 | -9.797 | 2.475 | 3.186 | 0 | 0.003 | 0.339 | NU |
| pLD | TP3 | m301 | Bacteria | Firmicutes | Clostridia | Clostridiales | Peptostreptococcaceae | Peptostreptococcus | 809 | -2.386 | 0.539 | 2.611 | 0 | 0.001 | 0.043 | NU |
| pLD | TP3 | m325 | Bacteria | Firmicutes | Clostridia | Clostridiales | Lachnospiraceae | Roseburia | 809 | 1.632 | 0.427 | 5.19 | 0 | 0.005 | 0.594 | NU |
| pLD | TP3 | m326 | Bacteria | Firmicutes | Clostridia | Clostridiales | Lachnospiraceae | Pseudobutyrivibrio | 809 | 1.657 | 0.4 | 3.607 | 0 | 0.002 | 0.152 | NU |
| pLD | TP3 | m333 | Bacteria | Bacteroidetes | Bacteroidia | Bacteroidales | Porphyromonadaceae | Parabacteroides | 809 | -5.677 | 1.23 | 2.979 | 0 | 0 | 0.018 | NU |
| pLD | TP3 | m3396 | Bacteria | Actinobacteria | Actinobacteria | Actinomycetales | Micrococcaceae | Rothia | 809 | -4.325 | 1.188 | 1.962 | 0 | 0.008 | 1 | NU |
| pLD | TP3 | m34303 | Bacteria | Bacteroidetes | Bacteroidia | Bacteroidales | Bacteroidaceae | Bacteroides | 809 | -2.757 | 0.732 | 1.923 | 0 | 0.006 | 0.748 | NU |
| pLD | TP3 | m365 | Bacteria | Bacteroidetes | Bacteroidia | Bacteroidales | Prevotellaceae | Alloprevotella | 809 | -9.627 | 2.155 | 3.34 | 0 | 0.001 | 0.036 | NU |
| pLD | TP3 | m3949 | Bacteria | Fusobacteria | Fusobacteriia | Fusobacteriales | Fusobacteriaceae | Fusobacterium | 809 | -9.038 | 2.18 | 3.354 | 0 | 0.002 | 0.153 | NU |
| pLD | TP3 | m44 | Bacteria | Proteobacteria | Epsilonproteobacteria | Campylobacterales | Campylobacteraceae | Campylobacter | 809 | -1.5 | 0.331 | 6.457 | 0 | 0.001 | 0.026 | NU |
| pLD | TP3 | m45649 | Bacteria | Proteobacteria | Gammaproteobacteria | Enterobacteriales | Enterobacteriaceae | Escherichia/Shigella | 809 | -2.039 | 0.527 | 1.971 | 0 | 0.004 | 0.486 | NU |
| pLD | TP3 | m463 | Bacteria | Firmicutes | Clostridia | Clostridiales | Ruminococcaceae | Intestinimonas | 809 | -5.983 | 1.148 | 3.888 | 0 | 0 | 0.001 | NU |
| pLD | TP3 | m477 | Bacteria | Firmicutes | Clostridia | Clostridiales | Peptococcaceae 1 | Peptococcus | 809 | -1.613 | 0.368 | 3.128 | 0 | 0.001 | 0.053 | NU |
| pLD | TP3 | m478 | Bacteria | Firmicutes | Clostridia | Clostridiales | Incertae Sedis XI | Murdochiella | 809 | -1.757 | 0.436 | 2.514 | 0 | 0.002 | 0.256 | NU |
| pLD | TP3 | m4827 | Bacteria | Bacteroidetes | Bacteroidia | Bacteroidales | Porphyromonadaceae | Porphyromonas | 809 | -3.65 | 0.97 | 2.022 | 0 | 0.006 | 0.76 | NU |
| pLD | TP3 | m53 | Bacteria | Proteobacteria | Gammaproteobacteria | Aeromonadales | Succinivibrionaceae | Succinivibrio | 809 | -2.083 | 0.478 | 10.566 | 0 | 0.001 | 0.059 | NU |
| pLD | TP3 | m530 | Bacteria | Firmicutes | Clostridia | Clostridiales | Clostridiales_Incertae Sedis XI | Parvimonas | 809 | -4.376 | 0.924 | 3.123 | 0 | 0 | 0.01 | NU |
| pLD | TP3 | m535 | Bacteria | Actinobacteria | Actinobacteria | Bifidobacteriales | Bifidobacteriaceae | Bifidobacterium | 809 | -3.634 | 0.947 | 2.136 | 0 | 0.004 | 0.56 | NU |
| pLD | TP3 | m561 | Bacteria | Proteobacteria | Epsilonproteobacteria | Campylobacterales | Helicobacteraceae | Helicobacter | 809 | -3.115 | 0.857 | 1.772 | 0 | 0.008 | 1 | NU |
| pLD | TP3 | m567 | Bacteria | Fusobacteria | Fusobacteriia | Fusobacteriales | Fusobacteriaceae | Fusobacterium | 809 | -4.745 | 1.289 | 2.07 | 0 | 0.007 | 1 | NU |
| pLD | TP3 | m628 | Bacteria | Firmicutes | Clostridia | Clostridiales | Lachnospiraceae | Roseburia | 809 | 1.85 | 0.415 | 4.671 | 0 | 0.001 | 0.038 | NU |
| pLD | TP3 | m638 | Bacteria | Bacteroidetes | Bacteroidia | Bacteroidales | Bacteroidaceae | Bacteroides | 809 | -6.903 | 1.574 | 2.864 | 0 | 0.001 | 0.052 | NU |
| pLD | TP3 | m647 | Bacteria | Firmicutes | Erysipelotrichia | Erysipelotrichales | Erysipelotrichaceae | Erysipelotrichaceae_incertae_sedis | 809 | -6.5 | 1.722 | 2.341 | 0 | 0.005 | 0.721 | NU |
| pLD | TP3 | m664 | Bacteria | Firmicutes | Clostridia | Clostridiales | Clostridiales_Incertae Sedis XI | Anaerococcus | 809 | -1.699 | 0.378 | 2.97 | 0 | 0.001 | 0.031 | NU |
| pLD | TP3 | m665 | Bacteria | Bacteroidetes | Bacteroidia | Bacteroidales | Rikenellaceae | Alistipes | 809 | -7.449 | 1.476 | 3.757 | 0 | 0 | 0.002 | NU |
| pLD | TP3 | m688 | Bacteria | Firmicutes | Clostridia | Clostridiales | Ruminococcaceae | Ruminococcus | 809 | -2.526 | 0.563 | 2.744 | 0 | 0.001 | 0.032 | NU |
| pLD | TP3 | m743 | Bacteria | Firmicutes | Clostridia | Clostridiales | Lachnospiraceae | Howardella | 809 | -7.629 | 1.844 | 3.026 | 0 | 0.002 | 0.158 | NU |
| pLD | TP3 | m82 | Bacteria | Firmicutes | Bacilli | Lactobacillales | Enterococcaceae | Enterococcus | 809 | -4.031 | 0.756 | 3.776 | 0 | 0 | 0 | NU |
| pLD | TP3 | m836 | Bacteria | Bacteroidetes | Bacteroidia | Bacteroidales | Bacteroidaceae | Bacteroides | 809 | -6.172 | 1.107 | 4.267 | 0 | 0 | 0 | NU |
| pLD | TP3 | m977 | Bacteria | Actinobacteria | Actinobacteria | Coriobacteriales | Coriobacteriaceae | Atopobium | 809 | -3.575 | 0.814 | 2.626 | 0 | 0.001 | 0.05 | NU |
| pLD | TP3 | m994 | Bacteria | Synergistetes | Synergistia | Synergistales | Synergistaceae | Cloacibacillus | 809 | -7.143 | 1.961 | 2.264 | 0 | 0.008 | 1 | NU |
| cADG2 | TP1 | m50608 | Bacteria | Firmicutes | Bacilli | Lactobacillales | Lactobacillaceae | Lactobacillus | 1190 | -3.475 | 0.843 | 1.086 | 0 | 0.008 | 0.168 | TE |
| cBF | TP1 | m39 | Bacteria | Firmicutes | Clostridia | Clostridiales | Lachnospiraceae | Coprococcus | 1183 | 0.664 | 0.179 | 0.853 | 0 | 0.006 | 0.905 | TE |
| cBF | TP1 | m468 | Bacteria | Firmicutes | Negativicutes | Selenomonadales | Veillonellaceae | Dialister | 1183 | 0.662 | 0.185 | 0.804 | 0 | 0.009 | 1 | TE |
| cBF | TP1 | m738 | Bacteria | Firmicutes | Clostridia | Clostridiales | Peptostreptococcaceae | Clostridium XI | 1183 | -1.244 | 0.353 | 0.75 | 0 | 0.01 | 1 | TE |
| cADG2 | TP2 | m2559 | Bacteria | Firmicutes | Bacilli | Lactobacillales | Lactobacillaceae | Lactobacillus | 1198 | 1.276 | 0.289 | 1.293 | 0 | 0.003 | 0.046 | TE |
| cADG2 | TP2 | m7245 | Bacteria | Firmicutes | Bacilli | Lactobacillales | Lactobacillaceae | Lactobacillus | 1198 | 1.921 | 0.429 | 1.235 | 0 | 0.003 | 0.034 | TE |
| cBEL | TP2 | m147 | Bacteria | Firmicutes | Clostridia | Clostridiales | Peptococcaceae 1 | Peptococcus | 1178 | 1.459 | 0.266 | 2.573 | 0 | 0 | 0 | TE |
| cBEL | TP2 | m560 | Bacteria | Firmicutes | Clostridia | Clostridiales | Peptococcaceae 1 | Peptococcus | 1178 | 1.511 | 0.263 | 2.799 | 0 | 0 | 0 | TE |
| cBF | TP2 | m1011 | Bacteria | Firmicutes | Negativicutes | Selenomonadales | Veillonellaceae | Selenomonas | 1191 | -1.266 | 0.357 | 0.854 | 0 | 0.009 | 1 | TE |
| cBF | TP2 | m1377 | Bacteria | Firmicutes | Bacilli | Lactobacillales | Lactobacillaceae | Lactobacillus | 1191 | 1.025 | 0.267 | 0.994 | 0 | 0.004 | 0.552 | TE |
| cBF | TP2 | m1468 | Bacteria | Firmicutes | Bacilli | Lactobacillales | Lactobacillaceae | Lactobacillus | 1191 | 1.029 | 0.278 | 0.942 | 0 | 0.006 | 0.952 | TE |
| cBF | TP2 | m147 | Bacteria | Firmicutes | Clostridia | Clostridiales | Peptococcaceae 1 | Peptococcus | 1191 | 1.253 | 0.244 | 1.886 | 0 | 0 | 0.001 | TE |
| cBF | TP2 | m1586 | Bacteria | Firmicutes | Clostridia | Clostridiales | Clostridiaceae 1 | Clostridium sensu stricto | 1191 | -1.009 | 0.283 | 0.86 | 0 | 0.009 | 1 | TE |
| cBF | TP2 | m1641 | Bacteria | Firmicutes | Bacilli | Lactobacillales | Lactobacillaceae | Lactobacillus | 1191 | 1.177 | 0.292 | 1.039 | 0 | 0.002 | 0.244 | TE |
| cBF | TP2 | m169 | Bacteria | Firmicutes | Clostridia | Clostridiales | Lachnospiraceae | Lachnospiracea_incertae_sedis | 1191 | 1.184 | 0.26 | 1.522 | 0 | 0.001 | 0.024 | TE |
| cBF | TP2 | m17 | Bacteria | Firmicutes | Bacilli | Lactobacillales | Lactobacillaceae | Lactobacillus | 1191 | 1.032 | 0.246 | 1.335 | 0 | 0.002 | 0.119 | TE |
| cBF | TP2 | m175 | Bacteria | Firmicutes | Clostridia | Clostridiales | Ruminococcaceae | Intestinimonas | 1191 | -0.854 | 0.223 | 1.018 | 0 | 0.005 | 0.59 | TE |
| cBF | TP2 | m1872 | Bacteria | Firmicutes | Bacilli | Lactobacillales | Lactobacillaceae | Lactobacillus | 1191 | 6.251 | 1.683 | 0.897 | 0 | 0.006 | 0.918 | TE |
| cBF | TP2 | m1902 | Bacteria | Firmicutes | Bacilli | Lactobacillales | Lactobacillaceae | Lactobacillus | 1191 | 1.087 | 0.27 | 1.059 | 0 | 0.002 | 0.25 | TE |
| cBF | TP2 | m200 | Bacteria | Bacteroidetes | Bacteroidia | Bacteroidales | Porphyromonadaceae | Parabacteroides | 1191 | -1.006 | 0.277 | 0.947 | 0 | 0.007 | 1 | TE |
| cBF | TP2 | m222 | Bacteria | Firmicutes | Clostridia | Clostridiales | Lachnospiraceae | Lachnospiracea_incertae_sedis | 1191 | 1.3 | 0.28 | 1.528 | 0 | 0.001 | 0.016 | TE |
| cBF | TP2 | m23 | Bacteria | Firmicutes | Clostridia | Clostridiales | Ruminococcaceae | Faecalibacterium | 1191 | 1.175 | 0.277 | 1.272 | 0 | 0.001 | 0.1 | TE |
| cBF | TP2 | m275 | Bacteria | Firmicutes | Clostridia | Clostridiales | Ruminococcaceae | Ruminococcus | 1191 | -1.047 | 0.279 | 0.934 | 0 | 0.006 | 0.803 | TE |
| cBF | TP2 | m28987 | Bacteria | Firmicutes | Bacilli | Lactobacillales | Lactobacillaceae | Lactobacillus | 1191 | 1.048 | 0.244 | 1.416 | 0 | 0.001 | 0.08 | TE |
| cBF | TP2 | m3143 | Bacteria | Firmicutes | Erysipelotrichia | Erysipelotrichales | Erysipelotrichaceae | Turicibacter | 1191 | 0.972 | 0.25 | 0.931 | 0 | 0.004 | 0.467 | TE |
| cBF | TP2 | m33615 | Bacteria | Firmicutes | Bacilli | Lactobacillales | Lactobacillaceae | Lactobacillus | 1191 | 0.956 | 0.201 | 1.657 | 0 | 0 | 0.009 | TE |
| cBF | TP2 | m349 | Bacteria | Bacteroidetes | Bacteroidia | Bacteroidales | Prevotellaceae | Prevotella | 1191 | -1.49 | 0.406 | 0.913 | 0 | 0.007 | 1 | TE |
| cBF | TP2 | m3565 | Bacteria | Firmicutes | Clostridia | Clostridiales | Clostridiaceae 1 | Clostridium sensu stricto | 1191 | -1.052 | 0.243 | 1.188 | 0 | 0.001 | 0.064 | TE |
| cBF | TP2 | m3603 | Bacteria | Firmicutes | Bacilli | Lactobacillales | Streptococcaceae | Streptococcus | 1191 | 0.998 | 0.242 | 1.104 | 0 | 0.002 | 0.162 | TE |
| cBF | TP2 | m366 | Bacteria | Spirochaetes | Spirochaetia | Spirochaetales | Spirochaetaceae | Treponema | 1191 | -0.813 | 0.197 | 1.209 | 0 | 0.002 | 0.167 | TE |
| cBF | TP2 | m416 | Bacteria | Bacteroidetes | Bacteroidia | Bacteroidales | Prevotellaceae | Prevotella | 1191 | -1.227 | 0.291 | 1.198 | 0 | 0.002 | 0.11 | TE |
| cBF | TP2 | m48470 | Bacteria | Firmicutes | Bacilli | Lactobacillales | Streptococcaceae | Streptococcus | 1191 | 1.191 | 0.294 | 1.152 | 0 | 0.002 | 0.235 | TE |
| cBF | TP2 | m486 | Bacteria | Firmicutes | Clostridia | Clostridiales | Ruminococcaceae | Ruminococcus | 1191 | 1.023 | 0.272 | 0.984 | 0 | 0.005 | 0.774 | TE |
| cBF | TP2 | m52033 | Bacteria | Firmicutes | Bacilli | Lactobacillales | Lactobacillaceae | Lactobacillus | 1191 | 0.967 | 0.248 | 1.155 | 0 | 0.004 | 0.423 | TE |
| cBF | TP2 | m55 | Bacteria | Firmicutes | Clostridia | Clostridiales | Ruminococcaceae | Butyricicoccus | 1191 | 0.889 | 0.192 | 1.54 | 0 | 0.001 | 0.017 | TE |
| cBF | TP2 | m560 | Bacteria | Firmicutes | Clostridia | Clostridiales | Peptococcaceae 1 | Peptococcus | 1191 | 1.337 | 0.236 | 2.201 | 0 | 0 | 0 | TE |
| cBF | TP2 | m595 | Bacteria | Firmicutes | Clostridia | Clostridiales | Lachnospiraceae | Blautia | 1191 | 1.043 | 0.273 | 0.962 | 0 | 0.005 | 0.6 | TE |
| cBF | TP2 | m601 | Bacteria | Firmicutes | Clostridia | Clostridiales | Lachnospiraceae | Coprococcus | 1191 | 1.022 | 0.27 | 0.958 | 0 | 0.005 | 0.696 | TE |
| cBF | TP2 | m8 | Bacteria | Bacteroidetes | Bacteroidia | Bacteroidales | Prevotellaceae | Prevotella | 1191 | 0.924 | 0.261 | 0.86 | 0 | 0.009 | 1 | TE |
| cBF | TP2 | m801 | Bacteria | Proteobacteria | Gammaproteobacteria | Aeromonadales | Succinivibrionaceae | Succinivibrio | 1191 | -1.513 | 0.326 | 1.431 | 0 | 0.001 | 0.016 | TE |
| cBF | TP2 | m88 | Bacteria | Firmicutes | Clostridia | Clostridiales | Lachnospiraceae | Coprococcus | 1191 | 1.308 | 0.363 | 0.879 | 0 | 0.008 | 1 | TE |
| cBF | TP2 | m9 | Bacteria | Firmicutes | Bacilli | Lactobacillales | Lactobacillaceae | Lactobacillus | 1191 | 0.93 | 0.202 | 1.542 | 0 | 0.001 | 0.018 | TE |
| cBF | TP2 | m910 | Bacteria | Firmicutes | Bacilli | Lactobacillales | Streptococcaceae | Streptococcus | 1191 | 0.923 | 0.248 | 0.943 | 0 | 0.006 | 0.912 | TE |
| cHAM | TP2 | m147 | Bacteria | Firmicutes | Clostridia | Clostridiales | Peptococcaceae 1 | Peptococcus | 1178 | -1.309 | 0.26 | 2.071 | 0 | 0.001 | 0.002 | TE |
| cHAM | TP2 | m560 | Bacteria | Firmicutes | Clostridia | Clostridiales | Peptococcaceae 1 | Peptococcus | 1178 | -1.463 | 0.254 | 2.626 | 0 | 0 | 0 | TE |
| cIMF | TP2 | m226 | Bacteria | Spirochaetes | Spirochaetia | Spirochaetales | Spirochaetaceae | Treponema | 1164 | -1.214 | 0.279 | 1.574 | 0 | 0.009 | 0.062 | TE |
| cIMF | TP2 | m398 | Bacteria | Bacteroidetes | Bacteroidia | Bacteroidales | Prevotellaceae | Prevotella | 1164 | 0.904 | 0.188 | 1.857 | 0 | 0.002 | 0.007 | TE |
| cIMF | TP2 | m9906 | Bacteria | Bacteroidetes | Bacteroidia | Bacteroidales | Prevotellaceae | Prevotella | 1164 | 1.368 | 0.285 | 1.816 | 0 | 0.002 | 0.007 | TE |
| cADG2 | TP3 | m1 | Bacteria | Firmicutes | Bacilli | Lactobacillales | Streptococcaceae | Streptococcus | 1181 | 1.097 | 0.265 | 1.795 | 0 | 0.008 | 0.161 | TE |
| cADG2 | TP3 | m122 | Bacteria | Firmicutes | Bacilli | Lactobacillales | Streptococcaceae | Streptococcus | 1181 | -1.016 | 0.25 | 1.084 | 0 | 0.009 | 0.222 | TE |
| cADG2 | TP3 | m147 | Bacteria | Firmicutes | Clostridia | Clostridiales | Peptococcaceae 1 | Peptococcus | 1181 | 1.86 | 0.309 | 2.581 | 0 | 0 | 0 | TE |
| cADG2 | TP3 | m1891 | Bacteria | Firmicutes | Clostridia | Clostridiales | Clostridiales_Incertae Sedis XI | Anaerococcus | 1181 | -1.247 | 0.281 | 1.295 | 0 | 0.003 | 0.042 | TE |
| cADG2 | TP3 | m33615 | Bacteria | Firmicutes | Bacilli | Lactobacillales | Lactobacillaceae | Lactobacillus | 1181 | 0.708 | 0.161 | 1.263 | 0 | 0.003 | 0.05 | TE |
| cADG2 | TP3 | m470 | Bacteria | Bacteroidetes | Bacteroidia | Bacteroidales | Bacteroidaceae | Bacteroides | 1181 | -1.625 | 0.389 | 1.202 | 0 | 0.007 | 0.133 | TE |
| cADG2 | TP3 | m48088 | Bacteria | Firmicutes | Clostridia | Clostridiales | Clostridiaceae 1 | Clostridium sensu stricto | 1181 | 1.203 | 0.282 | 1.143 | 0 | 0.005 | 0.092 | TE |
| cADG2 | TP3 | m851 | Bacteria | Lentisphaerae | Oligosphaeria | Oligosphaerales | Oligosphaeraceae | Oligosphaera | 1181 | -1.434 | 0.309 | 1.351 | 0 | 0.002 | 0.016 | TE |
| cADG2 | TP3 | m90 | Bacteria | Firmicutes | Clostridia | Clostridiales | Clostridiales_Incertae Sedis XI | Anaerococcus | 1181 | -0.557 | 0.136 | 1.117 | 0 | 0.008 | 0.195 | TE |
| cADG2 | TP3 | m910 | Bacteria | Firmicutes | Bacilli | Lactobacillales | Streptococcaceae | Streptococcus | 1181 | 1.35 | 0.279 | 1.548 | 0 | 0.001 | 0.006 | TE |
| cBEL | TP3 | m147 | Bacteria | Firmicutes | Clostridia | Clostridiales | Peptococcaceae 1 | Peptococcus | 1161 | 3.584 | 0.337 | 9.573 | 0 | 0 | 0 | TE |
| cBEL | TP3 | m2023 | Bacteria | Firmicutes | Clostridia | Clostridiales | Ruminococcaceae | Butyricicoccus | 1161 | 1.54 | 0.328 | 1.849 | 0 | 0.002 | 0.012 | TE |
| cBEL | TP3 | m294 | Bacteria | Firmicutes | Clostridia | Clostridiales | Lachnospiraceae | Roseburia | 1161 | 0.994 | 0.235 | 1.476 | 0 | 0.007 | 0.105 | TE |
| cBEL | TP3 | m436 | Bacteria | Firmicutes | Clostridia | Clostridiales | Ruminococcaceae | Butyricicoccus | 1161 | 1.351 | 0.287 | 1.87 | 0 | 0.002 | 0.011 | TE |
| cBEL | TP3 | m470 | Bacteria | Bacteroidetes | Bacteroidia | Bacteroidales | Bacteroidaceae | Bacteroides | 1161 | -1.959 | 0.433 | 1.762 | 0 | 0.003 | 0.027 | TE |
| cBEL | TP3 | m48776 | Bacteria | Spirochaetes | Spirochaetia | Spirochaetales | Spirochaetaceae | Treponema | 1161 | -1.546 | 0.357 | 1.62 | 0 | 0.006 | 0.066 | TE |
| cBEL | TP3 | m688 | Bacteria | Firmicutes | Clostridia | Clostridiales | Ruminococcaceae | Ruminococcus | 1161 | -1.257 | 0.287 | 1.597 | 0 | 0.005 | 0.055 | TE |
| cBEL | TP3 | m80 | Bacteria | Firmicutes | Clostridia | Clostridiales | Ruminococcaceae | Butyricicoccus | 1161 | -1.187 | 0.275 | 1.535 | 0 | 0.006 | 0.072 | TE |
| cBF | TP3 | m10347 | Bacteria | Lentisphaerae | Oligosphaeria | Oligosphaerales | Oligosphaeraceae | Oligosphaera | 1174 | -1.089 | 0.251 | 1.21 | 0 | 0.001 | 0.065 | TE |
| cBF | TP3 | m1103 | Bacteria | Firmicutes | Bacilli | Lactobacillales | Lactobacillaceae | Lactobacillus | 1174 | 1.677 | 0.404 | 1.106 | 0 | 0.002 | 0.148 | TE |
| cBF | TP3 | m1240 | Bacteria | Bacteroidetes | Bacteroidia | Bacteroidales | Prevotellaceae | Prevotella | 1174 | 1.138 | 0.312 | 1.015 | 0 | 0.007 | 1 | TE |
| cBF | TP3 | m1334 | Bacteria | Bacteroidetes | Bacteroidia | Bacteroidales | Rikenellaceae | Alistipes | 1174 | -0.892 | 0.239 | 0.929 | 0 | 0.006 | 0.844 | TE |
| cBF | TP3 | m1336 | Bacteria | Firmicutes | Clostridia | Clostridiales | Ruminococcaceae | Ruminococcus | 1174 | 1.131 | 0.23 | 1.579 | 0 | 0 | 0.004 | TE |
| cBF | TP3 | m1377 | Bacteria | Firmicutes | Bacilli | Lactobacillales | Lactobacillaceae | Lactobacillus | 1174 | 1.477 | 0.381 | 0.967 | 0 | 0.004 | 0.471 | TE |
| cBF | TP3 | m147 | Bacteria | Firmicutes | Clostridia | Clostridiales | Peptococcaceae 1 | Peptococcus | 1174 | 2.935 | 0.304 | 6.434 | 0 | 0 | 0 | TE |
| cBF | TP3 | m182 | Bacteria | Bacteroidetes | Bacteroidia | Bacteroidales | Prevotellaceae | Prevotella | 1174 | 1.139 | 0.256 | 1.419 | 0 | 0.001 | 0.04 | TE |
| cBF | TP3 | m1891 | Bacteria | Firmicutes | Clostridia | Clostridiales | Clostridiales_Incertae Sedis XI | Anaerococcus | 1174 | -1.092 | 0.286 | 0.998 | 0 | 0.005 | 0.617 | TE |
| cBF | TP3 | m19 | Bacteria | Firmicutes | Clostridia | Clostridiales | Lachnospiraceae | Roseburia | 1174 | 0.98 | 0.204 | 1.656 | 0 | 0 | 0.007 | TE |
| cBF | TP3 | m192 | Bacteria | Firmicutes | Clostridia | Clostridiales | Ruminococcaceae | Ruminococcus | 1174 | 1.026 | 0.27 | 0.957 | 0 | 0.005 | 0.65 | TE |
| cBF | TP3 | m2023 | Bacteria | Firmicutes | Clostridia | Clostridiales | Ruminococcaceae | Butyricicoccus | 1174 | 1.511 | 0.296 | 1.782 | 0 | 0 | 0.002 | TE |
| cBF | TP3 | m224 | Bacteria | Spirochaetes | Spirochaetia | Spirochaetales | Spirochaetaceae | Treponema | 1174 | -1.218 | 0.236 | 1.878 | 0 | 0 | 0.001 | TE |
| cBF | TP3 | m23 | Bacteria | Firmicutes | Clostridia | Clostridiales | Ruminococcaceae | Faecalibacterium | 1174 | 0.735 | 0.201 | 0.998 | 0 | 0.007 | 1 | TE |
| cBF | TP3 | m23350 | Bacteria | Firmicutes | Bacilli | Lactobacillales | Lactobacillaceae | Lactobacillus | 1174 | 1.385 | 0.312 | 1.249 | 0 | 0.001 | 0.04 | TE |
| cBF | TP3 | m24863 | Bacteria | Bacteroidetes | Bacteroidia | Bacteroidales | Prevotellaceae | Prevotella | 1174 | 0.707 | 0.2 | 0.849 | 0 | 0.01 | 1 | TE |
| cBF | TP3 | m24889 | Bacteria | Firmicutes | Bacilli | Lactobacillales | Streptococcaceae | Streptococcus | 1174 | 1.188 | 0.31 | 0.987 | 0 | 0.005 | 0.571 | TE |
| cBF | TP3 | m2495 | Bacteria | Proteobacteria | Epsilonproteobacteria | Campylobacterales | Helicobacteraceae | Helicobacter | 1174 | -1.142 | 0.287 | 1.017 | 0 | 0.003 | 0.317 | TE |
| cBF | TP3 | m255 | Bacteria | Firmicutes | Clostridia | Clostridiales | Lachnospiraceae | Roseburia | 1174 | 1.249 | 0.241 | 1.846 | 0 | 0 | 0.001 | TE |
| cBF | TP3 | m26 | Bacteria | Bacteroidetes | Bacteroidia | Bacteroidales | Bacteroidaceae | Bacteroides | 1174 | -1.038 | 0.254 | 1.244 | 0 | 0.002 | 0.197 | TE |
| cBF | TP3 | m27249 | Bacteria | Firmicutes | Clostridia | Clostridiales | Ruminococcaceae | Faecalibacterium | 1174 | 0.917 | 0.237 | 1.078 | 0 | 0.004 | 0.478 | TE |
| cBF | TP3 | m2743 | Bacteria | Firmicutes | Clostridia | Clostridiales | Lachnospiraceae | Coprococcus | 1174 | -1.356 | 0.36 | 1.022 | 0 | 0.005 | 0.745 | TE |
| cBF | TP3 | m294 | Bacteria | Firmicutes | Clostridia | Clostridiales | Lachnospiraceae | Roseburia | 1174 | 1.257 | 0.213 | 2.373 | 0 | 0 | 0 | TE |
| cBF | TP3 | m31 | Bacteria | Bacteroidetes | Bacteroidia | Bacteroidales | Prevotellaceae | Prevotella | 1174 | 1.448 | 0.321 | 1.376 | 0 | 0.001 | 0.029 | TE |
| cBF | TP3 | m325 | Bacteria | Firmicutes | Clostridia | Clostridiales | Lachnospiraceae | Roseburia | 1174 | 1.451 | 0.279 | 1.838 | 0 | 0 | 0.001 | TE |
| cBF | TP3 | m33615 | Bacteria | Firmicutes | Bacilli | Lactobacillales | Lactobacillaceae | Lactobacillus | 1174 | 0.742 | 0.164 | 1.383 | 0 | 0.001 | 0.027 | TE |
| cBF | TP3 | m349 | Bacteria | Bacteroidetes | Bacteroidia | Bacteroidales | Prevotellaceae | Prevotella | 1174 | -3.105 | 0.73 | 1.189 | 0 | 0.001 | 0.096 | TE |
| cBF | TP3 | m436 | Bacteria | Firmicutes | Clostridia | Clostridiales | Ruminococcaceae | Butyricicoccus | 1174 | 1.431 | 0.26 | 2.102 | 0 | 0 | 0 | TE |
| cBF | TP3 | m470 | Bacteria | Bacteroidetes | Bacteroidia | Bacteroidales | Bacteroidaceae | Bacteroides | 1174 | -1.468 | 0.395 | 0.979 | 0 | 0.006 | 0.925 | TE |
| cBF | TP3 | m472 | Bacteria | Firmicutes | Clostridia | Clostridiales | Lachnospiraceae | Blautia | 1174 | 1.286 | 0.308 | 1.169 | 0 | 0.002 | 0.132 | TE |
| cBF | TP3 | m55 | Bacteria | Firmicutes | Clostridia | Clostridiales | Ruminococcaceae | Butyricicoccus | 1174 | 0.667 | 0.187 | 0.998 | 0 | 0.009 | 1 | TE |
| cBF | TP3 | m564 | Bacteria | Firmicutes | Clostridia | Clostridiales | Lachnospiraceae | Blautia | 1174 | 1.199 | 0.273 | 1.265 | 0 | 0.001 | 0.051 | TE |
| cBF | TP3 | m57 | Bacteria | Firmicutes | Clostridia | Clostridiales | Clostridiaceae 1 | Clostridium sensu stricto | 1174 | -0.687 | 0.179 | 1.013 | 0 | 0.004 | 0.542 | TE |
| cBF | TP3 | m595 | Bacteria | Firmicutes | Clostridia | Clostridiales | Lachnospiraceae | Blautia | 1174 | 1.874 | 0.464 | 1.148 | 0 | 0.002 | 0.243 | TE |
| cBF | TP3 | m608 | Bacteria | Firmicutes | Bacilli | Lactobacillales | Aerococcaceae | Aerococcus | 1174 | -1.226 | 0.318 | 1.077 | 0 | 0.004 | 0.525 | TE |
| cBF | TP3 | m619 | Bacteria | Bacteroidetes | Bacteroidia | Bacteroidales | Prevotellaceae | Prevotella | 1174 | 1.622 | 0.287 | 2.217 | 0 | 0 | 0 | TE |
| cBF | TP3 | m62 | Bacteria | Firmicutes | Negativicutes | Selenomonadales | Veillonellaceae | Anaerovibrio | 1174 | 0.84 | 0.236 | 0.856 | 0 | 0.009 | 1 | TE |
| cBF | TP3 | m628 | Bacteria | Firmicutes | Clostridia | Clostridiales | Lachnospiraceae | Roseburia | 1174 | 1.423 | 0.331 | 1.2 | 0 | 0.001 | 0.076 | TE |
| cBF | TP3 | m648 | Bacteria | Firmicutes | Clostridia | Clostridiales | Lachnospiraceae | Pseudobutyrivibrio | 1174 | 1.401 | 0.319 | 1.265 | 0 | 0.001 | 0.051 | TE |
| cBF | TP3 | m688 | Bacteria | Firmicutes | Clostridia | Clostridiales | Ruminococcaceae | Ruminococcus | 1174 | -1.568 | 0.261 | 2.47 | 0 | 0 | 0 | TE |
| cBF | TP3 | m796 | Bacteria | Firmicutes | Clostridia | Clostridiales | Ruminococcaceae | Oscillibacter | 1174 | 1.466 | 0.388 | 0.939 | 0 | 0.005 | 0.726 | TE |
| cBF | TP3 | m80 | Bacteria | Firmicutes | Clostridia | Clostridiales | Ruminococcaceae | Butyricicoccus | 1174 | -1.547 | 0.248 | 2.627 | 0 | 0 | 0 | TE |
| cBF | TP3 | m851 | Bacteria | Lentisphaerae | Oligosphaeria | Oligosphaerales | Oligosphaeraceae | Oligosphaera | 1174 | -1.514 | 0.313 | 1.499 | 0 | 0 | 0.006 | TE |
| cBF | TP3 | m88 | Bacteria | Firmicutes | Clostridia | Clostridiales | Lachnospiraceae | Coprococcus | 1174 | 0.868 | 0.203 | 1.369 | 0 | 0.001 | 0.083 | TE |
| cBF | TP3 | m9 | Bacteria | Firmicutes | Bacilli | Lactobacillales | Lactobacillaceae | Lactobacillus | 1174 | 0.737 | 0.172 | 1.224 | 0 | 0.001 | 0.083 | TE |
| cBF | TP3 | m90 | Bacteria | Firmicutes | Clostridia | Clostridiales | Clostridiales_Incertae Sedis XI | Anaerococcus | 1174 | -0.524 | 0.138 | 0.998 | 0 | 0.005 | 0.648 | TE |
| cBF | TP3 | m910 | Bacteria | Firmicutes | Bacilli | Lactobacillales | Streptococcaceae | Streptococcus | 1174 | 1.082 | 0.285 | 0.993 | 0 | 0.005 | 0.661 | TE |
| cBF | TP3 | m9183 | Bacteria | Firmicutes | Clostridia | Clostridiales | Ruminococcaceae | Ruminococcus | 1174 | 1.027 | 0.255 | 1.04 | 0 | 0.002 | 0.249 | TE |
| cBF | TP3 | m983 | Bacteria | Firmicutes | Clostridia | Clostridiales | Ruminococcaceae | Faecalibacterium | 1174 | 2.01 | 0.537 | 0.938 | 0 | 0.006 | 0.812 | TE |
| cHAM | TP3 | m10347 | Bacteria | Lentisphaerae | Oligosphaeria | Oligosphaerales | Oligosphaeraceae | Oligosphaera | 1161 | 1.296 | 0.27 | 1.71 | 0 | 0.001 | 0.007 | TE |
| cHAM | TP3 | m147 | Bacteria | Firmicutes | Clostridia | Clostridiales | Peptococcaceae 1 | Peptococcus | 1161 | -3.1 | 0.334 | 7.161 | 0 | 0 | 0 | TE |
| cHAM | TP3 | m688 | Bacteria | Firmicutes | Clostridia | Clostridiales | Ruminococcaceae | Ruminococcus | 1161 | 1.252 | 0.283 | 1.584 | 0 | 0.007 | 0.044 | TE |
| cIMF | TP3 | m147 | Bacteria | Firmicutes | Clostridia | Clostridiales | Peptococcaceae 1 | Peptococcus | 1148 | 2.07 | 0.345 | 3.205 | 0 | 0 | 0 | TE |
| cIMF | TP3 | m955 | Bacteria | Firmicutes | Clostridia | Clostridiales | Lachnospiraceae | Roseburia | 1148 | 2.342 | 0.52 | 1.552 | 0 | 0.006 | 0.031 | TE |
| cLOI | TP3 | m147 | Bacteria | Firmicutes | Clostridia | Clostridiales | Peptococcaceae 1 | Peptococcus | 1161 | -2.77 | 0.347 | 5.716 | 0 | 0 | 0 | TE |
